# Biofilm viscoelasticity and nutrient source location control biofilm growth rate, migration rate, and morphology in shear flow

**DOI:** 10.1038/s41598-021-95542-1

**Published:** 2021-08-09

**Authors:** Hoa Nguyen, Abraham Ybarra, Hakan Başağaoğlu, Orrin Shindell

**Affiliations:** 1 Department of Mathematics, Trinity University, San Antonio, TX 78212 USA; 2 Evolution Online LLC, San Antonio, TX 78292 USA; 3 Department of Physics and Astronomy, Trinity University, San Antonio, TX 78212 USA

**Keywords:** Biophysics, Physics, Mathematics and computing, Applied mathematics, Computational science

## Abstract

We present a numerical model to simulate the growth and deformation of a viscoelastic biofilm in shear flow under different nutrient conditions. The mechanical interaction between the biofilm and the fluid is computed using the Immersed Boundary Method with viscoelastic parameters determined a priori from measurements reported in the literature. Biofilm growth occurs at the biofilm-fluid interface by a stochastic rule that depends on the local nutrient concentration. We compare the growth, migration, and morphology of viscoelastic biofilms with a common relaxation time of 18 min over the range of elastic moduli 10–1000 Pa in different nearby nutrient source configurations. Simulations with shear flow and an upstream or a downstream nutrient source indicate that soft biofilms grow more if nutrients are downstream and stiff biofilms grow more if nutrients are upstream. Also, soft biofilms migrate faster than stiff biofilms toward a downstream nutrient source, and although stiff biofilms migrate toward an upstream nutrient source, soft biofilms do not. Simulations without nutrients show that on the time scale of several hours, soft biofilms develop irregular structures at the biofilm-fluid interface, but stiff biofilms deform little. Our results agree with the biophysical principle that biofilms can adapt to their mechanical and chemical environment by modulating their viscoelastic properties. We also compare the behavior of a purely elastic biofilm to a viscoelastic biofilm with the same elastic modulus of 50 Pa. We find that the elastic biofilm underestimates growth rates and downstream migration rates if the nutrient source is downstream, and it overestimates growth rates and upstream migration rates if the nutrient source is upstream. Future modeling can use our comparison to identify errors that can occur by simulating biofilms as purely elastic structures.

## Introduction

Biofilms are ubiquitous in nature but could be detrimental in industrial and medical applications. When planktonic microorganisms attach to a solid surface immersed in a fluid flow, they become sessile and develop into biofilms^1^. Corrosion in pipelines due to biofilm formation is costly to the oil and gas industry, water utilities, and power plants^2^. In water transported to consumers through pipelines, biofilms growing on pipe walls release bacterial cells that deteriorate water quality and alter the drinking water microbiome^3^. The metal surfaces of vascular stents used to increase the diameter of arteries and improve blood flow in medical patients^4^ are susceptible to biofilm formation, which could lead to inflammation, necrosis, and vessel rupture^5^. In the dairy industry, biofilms that form on the stainless steel surfaces of milk storage tanks and process lines increase the risk of microbial contamination of processed dairy products^6^. In all these industrial settings, biofilms grow in a fluid environment under different nutrient supply and flow conditions.

Biofilms are composed of microorganisms bound together by a matrix of extracellular polymeric substances (EPS) that are secreted by the cells^7,8^. The matrix responds to mechanical stress by exhibiting elastic strain and viscous creep^9–12^ and its chemical composition facilitates nutrient capture^13^. By modulating their viscoelasticity and chemical composition through genetic expression, biofilms are capable of adapting to their mechanical and chemical environments^14–17^. For example, biofilms are able to resist mechanical clearance in the lungs of cystic fibrosis patients by adjusting their polysaccharide production^18^ and resist the immune response in chronic wound patients by producing rhamnolipids from quorum sensing cells^19^. Numerical models that incorporate realistic biofilm properties have the potential to make quantitative predictions about the mechanical and chemical behavior of biofilms in complex environmental conditions. Such predictions could suggest ways to mitigate biofilm growth in industrial and medical settings.

Mathematical models with different levels of complexity have been developed to simulate biofilm growth and deformation in fluid environments (for comprehensive reviews, see Refs.^20–22^ and references therein). The models can be generally classified into two groups^22^: those that simulate deformation, and those that simulate growth. The Immersed Boundary Method^23^ (IBM) has been a popular method of simulating biofilm deformation because it easily captures mechanical interactions between immersed structures and fluids. Some of the IBM models represented biofilms as purely elastic structures of nodes connected by Stokes elements^24,25^. Other models accounted for biofilm viscosity by changing the fluid viscosity near elastic biofilms^26,27^ or by representing biofilms as nodes connected by viscoelastic Kelvin–Voigt elements^28^. However, none of these IBM models included growth. Furthermore, those that included viscosity did not exhibit a constant strain rate at long times, hence, they could not reproduce the viscoelastic relaxation times measured in experiments^9,10^. Another set of models used discrete stochastic rules to simulate growing biofilms as they consume nutrients from their environment^29–38^. These models, however, did not include biofilm deformation. For large-scale simulations of biofilm processes, continuum models are utilized to simulate the fluid flow, nutrient distribution, and the spatial expansion of biomass. Examples include phase-field approaches that simulate the growth of non-elastic viscous biofilms^39^ or the deformation of viscoelastic biofilms that do not grow^40^. Even though these continuum models can produce results that match elaborate experiments quantitatively, their formulations tend to be complicated and computationally challenging^22^. Hybrid models that combine discrete biofilm structures and stochastic growth schemes with continuum formulations of nutrient and fluid dynamics can be easy-to-implement and computationally efficient.

In this paper, we present a novel hybrid biofilm model to study how the mechanical properties of biofilms affect their interactions with different nutrient supply and fluid flow conditions in a microchannel. The Reynolds number (*Re*) ranges from 0 to $8 \times 10^{-4}$ due to low shear velocities in our simulations . The magnitude of *Re* in our simulations is comparable to slow flow conditions in some of the earlier biolfim studies^41,42^. The biofilm is represented as a structure of nodes connected by viscoelastic Maxwell elements with viscoelastic parameter values determined a priori from experimental measurements of shear modulus and relaxation time^10,43^. The 2D simulation is based on the IBM and utilizes the IBM solver in the open source IB2d software^44–46^. An advection-diffusion-reaction equation, coupled with the IBM, governs the nutrient concentration in the fluid. As the biofilm consumes nutrients, new nodes and viscoelastic elements are added to the biofilm-fluid interface by a stochastic rule that is based on local concentration values. Our model can be used to predict the growth rate, migration rate, and morphology of viscoelastic biofilms that result from the interplay between viscoelastic deformation from fluid shear stress and growth from nutrient consumption over several hours.

Our paper is organized into three sections. In the Methods section, we discuss our approach to IBM that enables an a priori determination of parameter values, and we describe our biofilm growth and reaction algorithm. In the Results section, we present results from simulations with biofilms in shear flow with different flow velocities and nutrient source configurations. First we compare the results between a purely elastic biofilm and a viscoelastic biofilm with the same elastic modulus. Then we present the results for viscoelastic biofilms with a common relaxation time of 18 min^10^ over the range of elastic moduli 10–1000 Pa^10,43^. In the Discussion, we summarize the significance of our methods for future modeling work and interpret our results in a biophysical context.

## Methods

### Numerical model

Biofilms immersed in fluid and attached to a surface deform in response to fluid flow as they grow by consuming nutrients from the fluid environment. The deformation response of the EPS matrix and embedded cells can be described by bulk viscoelastic material properties, and the nutrient response by spatiotemporal chemical kinetics. Here, we present a biofilm model to simulate the growth of a viscoelastic biofilm attached to the wall of a microchannel with fluid flowing through the channel and a nutrient source located along the wall.

We use the Immersed Boundary Method (IBM)^47^ to simulate the hydrodynamic interactions between water and a growing viscoelastic biofilm, with viscoelastic simulation parameters determined a priori from experimental data. The fluid is defined in fixed Eulerian Cartesian coordinates ${\mathbf {x}} = \left( x,y\right)$ and coupled to the viscoelastic biofilm dynamics defined in variable Lagrangian Cartesian coordinates ${\mathbf {X}}(t)=\left( X(t),Y(t)\right)$. The Lagrangian coordinates of the biofilm are treated as nodes in a triangular mesh where the connections represent viscoelastic elements that exert forces ${\mathbf {F}}^*({\mathbf {X}},t)$ on the nodes. In IBM, the forces exerted on the Lagrangian nodes are transformed into body forces ${\mathbf {f}}({\mathbf {x}},t)$ exerted on the surrounding fluid particles in the Eulerian coordinates. The fluid attains velocity ${\mathbf {u}}({\mathbf {x}},t)$ as a result of its interactions with the immersed structure and other external forces. The fluid velocity is then interpolated to the Lagrangian nodes of the biofilm giving them velocities ${\mathbf {U}}({\mathbf {X}},t)$ that match the local fluid velocities so that the no-slip boundary condition is imposed at the immersed structure-fluid interface.

The dynamics of the concentration $c({\mathbf {x}},t)$ of a nutrient affecting biofilm growth is governed by an advection-diffusion-reaction equation solved in the Eulerian coordinates with a reaction term $r({\mathbf {x}},t)$ describing the nutrient uptake by the biofilm. As the biofilm consumes nutrients from the surrounding fluid environment, it grows by adding nodes and triangles to the Lagrangian structure via a stochastic rule based on local growth rates that are determined by local nutrient concentrations. The new nodes are connected by viscoelastic elements with mechanical properties that represent cells embedded in the EPS matrix. In the following subsections, we outline the details of our approach.

### Immersed boundary method

The motion of an incompressible Newtonian fluid that interacts with immersed structures is governed by the Navier Stokes equations

1 $$\begin{aligned} \rho \left( \frac{\partial {\mathbf {u}}({\mathbf {x}},t)}{\partial t} + {\mathbf {u}}({\mathbf {x}},t) \cdot \nabla {\mathbf {u}}({\mathbf {x}},t) \right)&= -\nabla p({\mathbf {x}},t) + \mu \nabla ^2 {\mathbf {u}}({\mathbf {x}},t) + {\mathbf {f}}_{\text {ext}}({\mathbf {x}},t) + {\mathbf {f}}({\mathbf {x}},t), \end{aligned}$$

2 $$\begin{aligned} \nabla \cdot {\mathbf {u}}\left( {\mathbf {x}},t\right)&=0, \end{aligned}$$

where ${\mathbf {u}}$ is the fluid velocity, *p* is the pressure, $\rho$ is the fluid density, $\mu$ is the dynamic viscosity, ${\mathbf {x}}$ is position in the Eulerian coordinates, and *t* is time. The net body force exerted on the fluid has a contribution ${\mathbf {f}}$ from the interaction between the immersed structure and the fluid and a contribution ${\mathbf {f}}_{\text {ext}}$ arising from all other external forces.

The density of forces acting on the biofilm due to internal stresses, described in Lagrangian coordinates as ${\mathbf {F}}({\mathbf {X}},t)$ at a position ${\mathbf {X}}$, is expressed in Eulerian coordinates through the equation

3 $$\begin{aligned} {\mathbf {f}}({\mathbf {x}},t) = \int _{\mathscr {L}} {\mathbf {F}}({\mathbf {X}},t) \delta \left( {\mathbf {x}} - {\mathbf {X}}\right) \,d{\mathscr {L}}, \end{aligned}$$

where the integral is taken over the Lagrangian domain ${\mathscr {L}}$. The integral in Eq. (3) with the Dirac delta function kernal $\delta \left( {\mathbf {x}} - {\mathbf {X}}\right)$ transforms the force density distributed on the immersed Lagrangian structure into body forces that are distributed on the surrounding fluid in the Eulerian domain. When the no-slip boundary condition is enforced at the immersed structure-fluid interface, the immersed structure must move at the local fluid velocity. In this case, the velocity of the immersed structure is given by

4 $$\begin{aligned} {\mathbf {U}}({\mathbf {X}},t) = \int _{{\mathscr {E}}} {\mathbf {u}}({\mathbf {x}},t) \delta \left( {\mathbf {x}} - {\mathbf {X}} \right) \,d{\mathscr {E}}, \end{aligned}$$

where the integral is taken over the Eulerian fluid domain ${\mathscr {E}}$. The integral transform in Eq. (4) interpolates the velocity of the fluid in the Eulerian domain to the immersed structure in the Lagrangian domain.

We use the open-source software IB2d^44–46^ to solve Eqs. (1), (2), (3) and (4) with periodic boundary conditions imposed at the inlet and outlet of the fluid domain. The Eulerian domain is discretized into a set of points ${\mathbf {x}}_{ij} = (x_i,y_j)$ defined on a square grid with grid spacing *h*. The Lagrangian structure is discretized into a set of points ${\mathbf {X}}_m(t) = (X_m(t),Y_m(t))$ where the distance between points may change at each time step. Equations (3) and (4) are implemented numerically by using the regularized delta function^47^

5 $$\begin{aligned}&\delta _h \left( {\mathbf {x}} \right) =\frac{1}{h^2} \displaystyle \phi \left( \frac{x}{h}\right) \phi \left( \frac{y}{h}\right) , \end{aligned}$$

6 $$\begin{aligned}&\phi \left( {\tilde{r}}\right) = {\left\{ \begin{array}{ll} \frac{1}{8}\left( 3 - 2\left| {\tilde{r}}\right| + \sqrt{1 + 4\left| {\tilde{r}}\right| - 4\left| {\tilde{r}}\right| ^2}\right) &{} \left| {\tilde{r}}\right| \le 1,\\ \frac{1}{8}\left( 5 - 2\left| {\tilde{r}}\right| - \sqrt{-7 + 12\left| {\tilde{r}}\right| - 4\left| {\tilde{r}} \right| ^2}\right) &{} 1\le \left| {\tilde{r}}\right| \le 2, \\ 0 &{} 2 \le \left| {\tilde{r}} \right| , \end{array}\right. } \end{aligned}$$

which has compact support within a square of side length 2*h*.

### Viscoelastic biofilm-wall structure

The biofilm in our model consists of set of Lagrangian nodes connected as a triangular mesh. To model the mechanics of the biofilm, the connections between nodes are treated as viscoelastic elements. The elements may be either a Stokes element, which is a linear spring that obeys Hooke’s law, or a Maxwell element, which consists of a Hookean spring and a dashpot in series. The force acting on the Lagrangian node ${\mathbf {X}}_m$ due to the element connecting it to Lagrangian node ${\mathbf {X}}_{m'}$ is given by

7 $$\begin{aligned} {\mathbf {F}}^*_{mm'}= d_{mm'}(0)^2 E \left( \frac{|| {\mathbf {X}}_{m'} - {\mathbf {X}}_{m} ||}{d_{mm'} \left( t \right) } -1 \right) \frac{{\mathbf {X}}_{m'} - {\mathbf {X}}_{m}}{|| {\mathbf {X}}_{m'} - {\mathbf {X}}_{m} ||}, \end{aligned}$$

where *E* is the elastic (Young’s) modulus of the biofilm and $d_{mm'}(t)$ is the resting length of the element. If the elements are purely elastic springs, the resting length, $d_{mm'}(t)$, in Eq. (7) is set equal to the initial resting length, $d_{mm'}(0)$.

For viscoelastic elements, the resting length of the element, $d_{mm'}(t)$, varies in time as the dashpot stretches or compresses. The rate of change of the resting length, $\dot{d}_{mm} (t)$, is proportional to the magnitude of the spring force^48^ and is given by

8 $$\begin{aligned} \dot{d}_{mm'} (t) = \frac{E d_{mm'}(0) }{\eta } \left( \frac{|| {\mathbf {X}}_{m'} - {\mathbf {X}}_{m} ||}{d_{mm'} \left( t \right) } -1 \right) , \end{aligned}$$

where $\eta$ is the dashpot viscosity. In the limit $\eta \rightarrow \infty$ at finite *E*, $\dot{d}_{mm'}(t) \rightarrow 0$, thus the element connecting ${\mathbf {X}}_m$ and ${\mathbf {X}}_{m'}$ becomes a purely elastic spring.

The walls of the simulation channel are each composed of a linear chain of Lagrangian nodes of initially equal spacing. Neighboring nodes are connected by stiff springs and each node is tethered to its original position by a stiff spring. The strong spring forces act to hold the nodes in place and thus impose a no-slip boundary condition at the wall. The force exerted on wall node ${\mathbf {X}}_w$ by the spring connecting it to its neighbor ${\mathbf {X}}_{w'}$ is

9 $$\begin{aligned} {\mathbf {F}}^*_{ww'} = A\left( \left\| {\mathbf {X}}_{w'} - {\mathbf {X}}_{w}\right\| - d_w\right) \frac{{\mathbf {X}}_{w'}-{\mathbf {X}}_{w}}{\left\| {\mathbf {X}}_{w'} - {\mathbf {X}}_{w}\right\| }, \end{aligned}$$

where $d_w$ is the initial spacing between the wall nodes. The force exerted on a wall node ${\mathbf {X}}_w$ by the spring tethering it to its fixed initial position ${\mathbf {X}}_{0w}$ is

10 $$\begin{aligned} {\mathbf {F}}^*_{ww} = B\left( {\mathbf {X}}_{0w}-{\mathbf {X}}_{w}\right) . \end{aligned}$$

The biofilm nodes at the bottom of the biofilm are initially placed along the wall co-linearly with the wall nodes. For the node ${\mathbf {X}}_m$ located initially along the wall, a stiff tether force connects it to its initial position ${\mathbf {X}}_{0m}$

11 $$\begin{aligned} {\mathbf {F}}^{*(w)}_{mm} = C\left( {\mathbf {X}}_{0m} - {\mathbf {X}}_{m} \right) . \end{aligned}$$

In the simulation of the biofilm in fluid flow, the flow is allowed to develop into a steady-state before allowing the biofilm to deform. This is accomplished by temporarily tethering each biofilm node ${\mathbf {X}}_{m}$ to its initial position ${\mathbf {X}}_{0m}$ with a stiff force

12 $$\begin{aligned} {\mathbf {F}}^*_{mm} = D\left( {\mathbf {X}}_{0m} - {\mathbf {X}}_{m} \right) . \end{aligned}$$

Once the flow has fully developed, the tethering force (Eq. 12) is released, thereby allowing the biofilm to deform.

The spring constants appearing in Eqs. (9), (10), (11), and (12) are much stiffer than the springs connecting the biofilm nodes, i.e., $A,\,B,\,C,\,D\gg Ed$ with *d* a typical biofilm spring resting length.

The forces acting on the biofilm and wall nodes can be used to define a force denstity function on the biofilm-wall Lagrangian structure. With ${\mathbf {F}}^*_{m}$ the net viscoelastic force acting on biofilm node ${\mathbf {X}}_m$ (from Eqs. 7, 11, and 12) and ${\mathbf {F}}^*_{w}$ the net spring force acting on wall node ${\mathbf {X}}_w$ (from Eqs. 9, 10), the force density on the immersed structure is

13 $$\begin{aligned} {\mathbf {F}}({\mathbf {X}},t) = \sum _m{\mathbf {F}}^*_m\delta \left( {\mathbf {X}} - {\mathbf {X}}_m\right) + \sum _w{\mathbf {F}}^*_w\delta \left( {\mathbf {X}} - {\mathbf {X}}_w\right) , \end{aligned}$$

where the Dirac delta functions distribute the forces to the Lagrangian nodes at which they act. Inserting Eq. (13) into Eq. (3) gives the total body force acting on the fluid due to its interaction with the biofilm-wall structure as^26^

14 $$\begin{aligned} {\mathbf {f}}({\mathbf {x}},t) = \sum _m{\mathbf {F}}^*_m\delta \left( {\mathbf {x}} - {\mathbf {X}}_m\right) + \sum _w{\mathbf {F}}^*_w\delta \left( {\mathbf {x}} - {\mathbf {X}}_w\right) , \end{aligned}$$

which may be regularized on the discrete Eulerian grid via Eq. (5).

In Supplementary Information-1, we discuss the origin of Eq. (13) and the calculation that leads to Eq. (14) via Eq. (3). We also show the connection between this formalism, which allows an a priori determination of the viscoelastic parameter values *E* and $\eta$, and other approaches that define a force density on the immersed structure through a “stiffness constant” with dimensions of force density. In Supplementary Information-2 and -3, we verify the accuracy of Eq. (14) by simulating a creep test on the biofilm structure where the elastic modulus *E* and dashpot viscosity $\eta$ were derived directly from measured values of the shear modulus and relaxation time in biofilm experiments^10^. The creep test analysis indicates that the simulated elastic shear modulus and relaxation time are consistent with chosen experimental values.

### Chemical dynamics and biofilm growth model

The spatiotemporal dynamics of the nutrient concentration in a fluid enivronment is governed by an advection-diffusion-reaction equation,

15 $$\begin{aligned} \frac{\partial c({\mathbf {x}},t)}{\partial t} = -{\mathbf {u}}({\mathbf {x}},t) \cdot \nabla c({\mathbf {x}},t) + D_c\nabla ^2 c({\mathbf {x}},t) + r({\mathbf {x}},t) , \end{aligned}$$

where *c* is the concentration of a single nutrient expressed in Eulerian coordinates ${\mathbf {x}}$ and $D_c$ is the homogeneous diffusion constant assumed to be the same within and outside the biofilm. In the present model, the reaction term *r* accounts for the uptake of nutrient concentration defined in the Eulerian domain by the biofilm described as a Lagrangian immersed structure.

Bacterial growth resulting from nutrient consumption can be modeled using Monod kinetics^49,50^, which defines a specific bacterial growth rate as a function of nutrient concentration,

16 $$\begin{aligned} \Gamma ({\mathbf {X}},t) = \mu _{\text {max}} \frac{C({\mathbf {X}},t)}{K + C({\mathbf {X}},t)}, \end{aligned}$$

where $\mu _{\text {max}}$ is the maximum specific growth rate, *C* is the local nutrient concentration expressed in the Lagrangian coordinates, and *K* is the half saturation constant. The time scale of our simulations is much shorter than the characteristic time of bacterial cell death^51^, thus a decay term is not included in Eq. (16). The net biofilm mass increases in time according to

17 $$\begin{aligned} \frac{dM}{dt} = {\bar{\Gamma }}(t)M, \end{aligned}$$

where *M* is the total biomass and

18 $$\begin{aligned} {\bar{\Gamma }}(t) = \frac{1}{\int \limits _{\mathscr {L}}d{\mathscr {L}}} \int _{{\mathscr {L}}}\Gamma ({\mathbf {X}},t)d{\mathscr {L}}, \end{aligned}$$

is the specific growth rate averaged over the Lagrangian biofilm structure. The biomass accumulated during biofilm growth is distributed along the biofilm boundary according to a probability density function proportional to the local specific growth rate,

19 $$\begin{aligned} \psi ({\mathbf {X}},t) = {\left\{ \begin{array}{ll} \dfrac{\Gamma ({\mathbf {X}},t)}{\int \limits _{{\mathscr {B}}} \Gamma ({\mathbf {X}},t)d{\mathscr {B}}} &{} \hbox { for}\ {\mathbf {X}} \in {\mathscr {B}}, \\ 0 &{} \text {otherwise,} \end{array}\right. } \end{aligned}$$

where ${\mathscr {B}}$ is the portion of the Lagrangian biofilm boundary in contact with the surrounding fluid. As the biofilm grows it consumes nutrients within a region ${\mathscr {E}}_r$ of the Eulerian domain that coincides with the Lagrangian biofilm structure. The reaction term in Eq. (15) is thus given by

20 $$\begin{aligned} r({\mathbf {x}},t) = {\left\{ \begin{array}{ll} \dfrac{dm}{dt}\dfrac{\gamma ({\mathbf {x}},t)}{\int \limits _{{\mathscr {E}}_r} \gamma ({\mathbf {x}},t)d{\mathscr {E}}} &{} \text {for}\,{\mathbf {x}}\in {\mathscr {E}}_r,\\ 0 &{} \text {otherwise}, \end{array}\right. } \end{aligned}$$

where $\frac{dm}{dt}$ is the net rate of change in nutrient mass due to consumption by the biofilm and

21 $$\begin{aligned} \gamma {({\mathbf {x}},t)} = \mu _\text {max}\frac{c({\mathbf {x}},t)}{K + c({\mathbf {x}},t)} \end{aligned}$$

is the local specific growth rate expressed in Eulerian coordinates. Finally, the net rate of biomass accumulation is proportional to the net rate of nutrient consumption,

22 $$\begin{aligned} \frac{dM}{dt} = Y\left| \frac{dm}{dt}\right| , \end{aligned}$$

where $0\le Y\le 1$ is the yield coefficient. In the next subsection we describe our implementation of the Eqs. (15)–(22) within the IB2d software, where we modified the advection-diffusion upwind scheme to accommodate the reaction term.

### Growth and reaction algorithm

The growth and reaction algorithm begins by initializing the Lagrangian biofilm structure and the nutrient concentration values defined in the Eulerian fluid domain. The biofilm is constructed by specifying the semicircular shape of the biofilm with radius $r_c$ and the distance between biofilm nodes *ds*. Then the software package DistMesh^52^ is used to create a triangular mesh^25^ that consists of $Q=Q_0$ nodes, $N=N_0$ triangles of average area $a_0$, and $S = S_0$ connections (or edges). DistMesh uses the Delaunay triangulation algorithm to produce a mesh that contains mostly equilateral and uniform triangles. Existing nodes and the connections between them are maintained throughout the simulation, and additional nodes and connections are added during the biofilm growth as discussed below. The two-dimensional biofilm is taken to represent a $\chi = 1\mu \text {m}$-thick slice of a three-dimensional biofilm. Thus, the bacterial biomass assigned to an average triangle is $m_0 = \lambda v_0 n_0 \chi a_0$ where $\lambda$ is the mass density of a bacterium, $v_0$ is the volume of a bacterium, and $n_0$ is the number density of bacteria in a three-dimensional biofilm. The *m*th node of the biofilm Lagrangian structure has the coordinate ${\mathbf {X}}_m = (X_m,Y_m)$.

The concentration values are defined in the Eulerian fluid domain, which is discretized into a square grid with side length *h*. The grid point ${\mathbf {x}}_{ij} = (x_i,y_j)$ has the concentration value $c_{ij}$. The concentration value $C_m$ at each Lagrangian biofilm node ${\mathbf {X}}_m$ is determined by averaging the concentrations at the corners of the Eulerian grid square containing ${\mathbf {X}}_m$,

23 $$\begin{aligned} C_m = \frac{1}{4}\sum _{{\mathbf {x}}_{ij}\in I_m}c_{ij}, \end{aligned}$$

where $$I_m \equiv \left[ \max \left\{ x_{i}< X_m\right\} , \max \left\{ x_{i}< X_m\right\} + h\right] \times \left[ \max \left\{ y_{j}< Y_m\right\} , \max \left\{ y_{j} < Y_m\right\} + h\right]$$.

The concentration values on the Lagrangian biofilm structure can be used to compute the increase of biomass. The local specific growth rate, Eq. (16), is approximated as

24 $$\begin{aligned} \Gamma _m = \mu _\text {max}\frac{C_m}{K + C_m}, \end{aligned}$$

while the average specific growth rate, Eq. (18), becomes

25 $$\begin{aligned} {\bar{\Gamma }} = \frac{1}{Q}\sum _{m=1}^Q\Gamma _m. \end{aligned}$$

During a given time step of size $\delta t$, the incremental increase in biofilm mass $\delta M$ is found by solving Eq. (17) with ${\bar{\Gamma }}$ from Eq. (25) using the forward Euler method,

26 $$\begin{aligned} \delta M = {{\bar{\Gamma }}} Nm_0 \delta t, \end{aligned}$$

where $Nm_0 = M$ is the mass of the biofilm at the beginning of the time step.

As the biofilm mass increases, the numbers of nodes and triangles increase. For each increase of biofilm mass $\delta M$, the increase in the triangle number is $\delta N = \delta M/m_0$. At the end of each time step, the change in triangle number accumulates from $\Delta N$ to $\Delta N + \delta N$. A number $N' = \lfloor \Delta N + \delta N\rfloor$ new triangles of area $a_0$ are then spawned at the boundary of the biofilm and the triangle accumulation is reset to the residue $\Delta N + \delta N - N'$. The growth of new triangles involves the addition of $Q'$ new nodes. Each new triangle is added to the biofilm boundary using the probabilistic growth rule, Eq. (19), discretized as

27 $$\begin{aligned} p_m = {\left\{ \begin{array}{ll} \dfrac{\Gamma _m}{\sum \limits _{{\mathbf {X}}_m\in {\mathscr {B}}}\Gamma _m} &{} \text {for }{\mathbf {X}}_m\in {\mathscr {B}},\\ 0 &{} \text {otherwise}, \end{array}\right. } \end{aligned}$$

where $p_m$ is the probability the new triangle will be spawned at ${\mathbf {X}}_m$. A position ${\mathbf {X}}_{m'}$ is chosen as one vertex of a candidate triangle (which is checked against conditions discussed below) by generating a random number $\xi$ distributed uniformly on the interval (0, 1) and choosing an integer $m'$ to satisfy

28 $$\begin{aligned} \sum \limits _{m = 1}^{m'-1}p_m< \xi < \sum \limits _{m=1}^{m'}p_m . \end{aligned}$$

Once the position ${\mathbf {X}}_{m'}$ is selected, a second boundary point ${\mathbf {X}}_{m''}$ adjacent to ${\mathbf {X}}_{m'}$ in the clockwise direction is selected (unless ${\mathbf {X}}_{m'}$ is the last clockwise boundary node, then ${\mathbf {X}}_{m''}$ is adjacent in the counterclockwise direction). A third point ${\mathbf {X}}'$ is then computed as the point located along the bisector to the line joining ${\mathbf {X}}_{m'}$ and ${\mathbf {X}}_{m''}$ such that the area of the isosceles triangle joining ${\mathbf {X}}_{m'}$, ${\mathbf {X}}_{m''}$, and ${\mathbf {X}}'$ is $a_0$. If ${\mathbf {X}}'$ is within a small distance $\varepsilon$ from an existing biofilm boundary point ${\mathbf {X}}_{m'''}$, then a new triangle is formed by connecting ${\mathbf {X}}_{m'}$, ${\mathbf {X}}_{m''}$, and ${\mathbf {X}}_{m'''}$. If the candidate triangle overlaps the existing biofilm but the third point ${\mathbf {X}}'$ is not within $\varepsilon$ of an existing point, or if the triangle overlaps the wall, a different position is chosen as the vertex of a new candidate triangle via Eq. (28). If neither of the former two conditions hold, a point ${\mathbf {X}}_{Q + n}={\mathbf {X}}'$, where $n=1,\cdots ,Q'$, is spawned and connected to the two boundary points ${\mathbf {X}}_{m'}$ and ${\mathbf {X}}_{m''}$ to form a new triangle.

After each time step, the biofilm mass increases by an incremental amount $\delta M$ and it consumes an incremental mass of nutrient $\delta m = \frac{\delta M}{Y}$. The nutrient is consumed within the region ${\mathscr {E}}_r$, which is computed as a polygonal region of the Eulerian domain that tightly encloses the biofilm Lagrangian structure using the MATLAB function ‘polyshape’. The local specific growth rate defined in the Eulerian domain is discretized as

29 $$\begin{aligned} \gamma _{ij} = \mu _{\text {max}}\frac{c_{ij}}{K + c_{ij}}. \end{aligned}$$

The discrete form of the reaction term Eq. (20) is thus

30 $$\begin{aligned} r_{ij} = {\left\{ \begin{array}{ll} -\dfrac{1}{h^2}\dfrac{1}{Y}\dfrac{\delta M}{\delta t} \dfrac{\gamma _{ij}}{\sum \limits _{{\mathbf {x}}_{ij}\in {\mathscr {E}}_r} \gamma _{ij}} &{} \hbox { for}\ {\mathbf {x}}_{ij}\in {\mathscr {E}}_r, \\ 0 &{} \text {otherwise,} \end{array}\right. } \end{aligned}$$

where *h* is the Eulerian grid size and $\delta t$ is the time step.

The growth and reaction algorithm can be summarized as follows: At the initial time $t = t_0$, the biofilm structure is discretized as a triangular mesh containing $Q=Q_0$ nodes located at positions $\left\{ {\mathbf {X}}_m\right\} = \left\{ {\mathbf {X}}_{0m}\right\}$, $S=S_0$ connections, and $N = N_0$ triangles with the triangle accumulation $\Delta N = 0$ and the biomass assigned to an average triangle $m_0$. The concentration field $c_{ij}$ is also initialized in the Eulerian domain at the coordinates ${\mathbf {x}}_{ij}$. To update the biofilm mass and reaction term at time *t*, we perform the following process:

1. Given *Q*, $\left\{ {\mathbf {X}}_m\right\}$, *S*, *N*, $\Delta N$, and $c_{ij}$, use Eqs. (23)–(26) to compute the incremental increase in biofilm mass $\delta M$, and Eqs. (29) and (30) to calculate the reaction term $r_{ij}.$
2. Due to the increase of the biomass, a growing biofilm is formed by adding $N' = \lfloor \Delta N + \frac{\delta M}{m_0}\rfloor$ new triangles, $Q'$ new nodes located at points $\left\{ {\mathbf {X}}_{Q + n}\right\}$, and $S'$ new connections. Equations (27)–(28) present the strategy in forming these additional triangles.
3. Set $t\leftarrow t + \delta t$, $Q \leftarrow Q + Q'$, $\left\{ {\mathbf {X}}_m\right\} \leftarrow \left\{ {\mathbf {X}}_m\right\} \bigcup \left\{ {\mathbf {X}}_{Q+n}\right\}$, $S \leftarrow S + S'$, $N \leftarrow N + N'$, $\Delta N \leftarrow \Delta N + \frac{\delta M}{m_0} - N'$, and $c_{ij} \leftarrow c_{ij} + r_{ij}\delta t$. Return to Step 1 or end the simulation when the maximum number of time steps is reached.

The MATLAB code that couples our growth and reaction algorithm with the IBM solver in IB2d^44–46^ is included in the open source software package listed in the Supplementary Information-6. This package contains full implementation of the simulations presented in the next section and the creep tests.

## Results

### Numerical simulations

**Figure 1:**
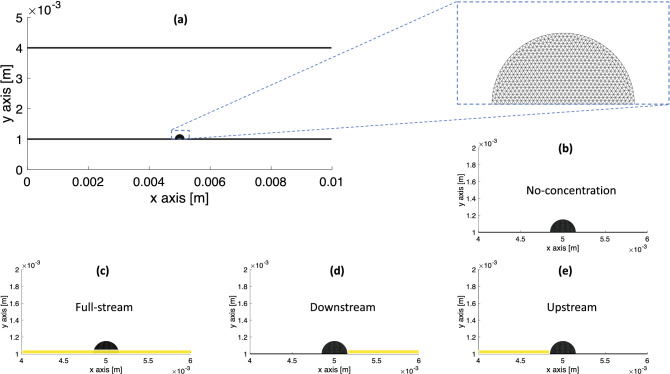
Initial simulation set up. (**a**) The initial configuration is a semicircular biofilm located at the bottom of the microchannel with a triangular mesh (inset) with $Q_0 = 637$ nodes, $S_0 = 1819$ viscoelastic elements, and $N_0 = 1183$ triangles. The periodic boundary condition is imposed at the inlet and outlet, and the no-slip boundary condition is implemented along the walls. (**b**) The base case of no-concentration used to simulate biofilm deformation in fluid flow without growth. (**c**)–(**e**) The test cases of concentration (yellow strip) to simulate growth under different nutrient conditions in the presence of fluid flow. During the simulation, the concentration within the yellow strip, which is one Eulerian grid width thick, is held constant at $c_0$ to simulate a continuous supply of nutrient.

We simulate the deformation and growth of elastic and viscoelastic biofilms of a single species biofilm already in the adhesion or proliferation stage^53^ in fluid flow with a nutrient source. The biofilms are attached to the wall of a 2D microchannel, which has two walls parallel to the *x*-axis constructed from Lagrangian wall nodes. The length of the channel walls is $L_x$ and the distance between the walls is $L_y$. A no-slip boundary condition on the walls is imposed by connecting adjacent nodes to each other and tethering each node to its initial position with stiff Stokes elements (Eqs. 9 and 10). Periodic boundary conditions are imposed at the inlet and outlet of the channel. The biofilm is initialized as a semicircular triangular-mesh structure located at the lower wall with $Q_0$ Lagrangian nodes, $S_0$ viscoelastic elements, and $N_0$ triangles. To anchor the biofilm to the channel wall, the biofilm nodes initially co-linear with the wall are tethered to their initial position with stiff Stokes elements (Eq. 11). The simulation set up is shown in Fig. 1a. The semicircular initial shape of the biolfim in Fig. 1 is consistent with the semicircular fan shape of *P. aerugionasa* biofilms on the polydimethylsiloxane template above the inoculation point observed from macroscopic scans^54^.

We compute the deformation of each biofilm over a range of fluid flow rates. The fluid flows are simulated on a square-grid Eulerian fluid domain with the grid size *h*. Each flow is imposed by applying an external body force ${\mathbf {F}}_\text {ext} = \rho g \hat{\mathbf{x}}$ to each fluid node, where $\rho$ is the fluid density, *g* is an acceleration constant, and $\hat{\mathbf{x}}$ is the *x*-direction unit vector. The acceleration constant *g* is determined using the steady-state 2D Poiseuille flow solution such that a desired maximum fluid velocity $u_\text {max}$ occurs along the midchannel:

31 $$\begin{aligned} g = \frac{8\mu u_\text {max}}{\rho {L_y}^2}. \end{aligned}$$

To determine the accuracy of our flow, we first perform simulations in the microchannel without the biofilm structure by applying ${\mathbf {F}}_\text {ext}$ to each fluid node with *g* determined from Eq. (31). The simulated maximum fluid velocity reaches its steady state after 20 seconds and matches the desired maximum fluid velocity $u_\text {max}$ (listed in Table 1) within 2%. Therefore, in simulations with the biofilm structure present, the biofilm is tethered in place for the first 20 s of the simulation by stiff Stokes elements (Eq. 12) as the flow evolves into a steady-state. Then the tethers are released allowing the biofilm to deform in response to the shear flow.

We simulate the growth of each biofilm in different nutrient concentration configurations shown in Fig. 1b–e to account for the combined effect of the shear flow and nutrient source location on the growth of a viscoelastic or elastic biofilm. At 20 seconds the concentration is initialized as $c_0$ at the Eulerian grid points within the highlighted region of height *h* as indicated in Fig. 1c–e. During the simulation, the concentration is held constant at $c_0$ on the same Eulerian grid points to represent a continuous supply of nutrient. The base case of zero nutrient configuration Fig. 1b is used to test the deformation of the biofilm in the presence of flow but in the absence of growth. Then the growth and deformation of the biofilms are tested under the full-stream Fig. 1c, downstream Fig. 1d, and upstream Fig. 1e nutrient configurations. The numerical values of all simulation parameters are displayed in Table 1.

**Table 1:** Parameters used in numerical simulations.

Parameter	Symbol	Value	Unit	Reference
Dynamic viscosity of the fluid	$\mu$	$9.31\times 10^{-4}$	Pa.s	
Density of the fluid	$\rho$	1000	kg$/$ $\hbox {m}^3$	
Length of channel	$L_x$	$1 \times 10^{-2}$	m	
Width of channel	$L_y$	$3 \times 10^{-3}$	m	
Eulerian grid size	$dx = dy = h$	$4.17 \times 10^{-5}$	m	
Grid size on biofilm	*ds*	*dx*/5	m	
Time step	$\delta t$	$1 \times 10^{-3}$	s	
Elastic modulus of biofilm in Section:	*E*			^10,43^
*Elastic versus Viscoelastic Biofilms*		50	Pa	
*Viscoelastic Biofilms*		10, 50, 100, 500, 1000	Pa	
Dashpot viscosity (Stokes spring)	$\eta$	$\infty$	Pa.s	
Dashpot viscosity (Maxwell element) in Section:	$\eta$			
*Elastic versus Viscoelastic Biofilms*		$5 \times 10^{4}$	Pa.s	
*Viscoelastic Biofilms*		$b_d/ds$	Pa.s	(*)
Stiffness constant for connecting springs on wall	*A*	$8.33 \times 10^{-2}$	N/m	
Tethered-spring stiffness on wall	*B*	$6.25 \times 10^{-2}$	N/m	
Tethered-spring stiffness on biofilm bottom nodes	*C*	$6.25 \times 10^{-2}$	N/m	
Tethered-spring stiffness to freeze biofilm (first 20 s)	*D*	$8.33 \times 10^{-2}$	N/m	
Radius of initial biofilm semi-circle	$r_c$	$1.5 \times 10^{-4}$	m	
Maximum shear velocity	$u_{\text {max}}$	$0, 1 \times 10^{-6},\ldots ,5 \times 10^{-6}$	m/s	^35^
Reynolds number (${\rho r_c u_{\text {max}}}/{\mu }$)	*Re*	$0,\ldots ,8 \times 10^{-4}$		
Initial concentration of nutrient	$c_0$	$1.0\times 10^{-3}$	kg/$\hbox {m}^3$	^35^
Diffusion rate of nutrient	$D_c$	$1.0\times 10^{-10}$	$\hbox {m}^2/$s	^55^
Maximum specific growth rate of bacteria	$$\mu _{max}$$	$$2 \times 10^{-4}$$	$$\hbox {s}^{-1}$$	^35,56^$$^{\dagger }$$
Bacterial yield coefficient	*Y*	0.553	-	^57,58^$$^{\dagger }$$
Half saturation constant	*K*	$1\times 10^{-4}$	kg/$\hbox {m}^3$	^33,59^
Bacterial mass density	$\lambda$	1.12	$\mathrm {g}/\mathrm {ml}$	^60^
Volume of bacterium	$v_0$	1.75	${\mu \mathrm {m}}^{3}$	
Biofilm bacteria number density	$n_0$	0.21	${\mu \mathrm {m}}^{-3}$	^61^
Average area of initial triangles	$a_0$	$2.99\times 10^{-11}$	$\hbox {m}^2$	

(*) The calculation of the drag coefficient $b_d$ is shown at the end of Supplementary Information-2: Viscoelastic Parameters.

$(^{\dagger })$ These values are chosen within the range of the values reported in the corresponding references.

### Elastic versus viscoelastic biofilms

We use our model to compare the behavior of a purely elastic biofilm composed of Stokes elements to a more biologically realistic viscoelastic biofilm composed of Maxwell elements in a shear flow. The elastic modulus is chosen to be $E = 50$ Pa in both cases. The viscoelastic biofilm has a dashpot viscosity $\eta = 5\times 10^4$ Pa.s, which is chosen to give a relaxation time near the 18 min that is common in biofilms over a wide range of elastic moduli^10^. In Supplementary Information-3, the creep test on the viscoelastic biofilm structure is presented to show how the elastic modulus, dashpot parameter, and relaxation time are related.

**Figure 2:**
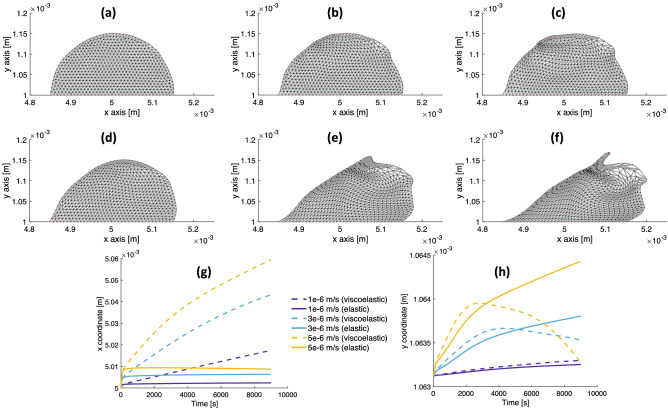
Comparison of deformation in shear flow between an elastic biofilm ($E = 50$ Pa) and a viscoelastic biofilm ($E = 50$ Pa, $\eta = 5\times 10^4$ Pa.s). (**a**)–(**f**) From top to bottom: elastic to viscoelastic; from left to right: $u_\text {max} = 1, 3, 5\times 10^{-6}$ m/s. Protrusions appear in viscoelastic biofilm at flow rates that are absent in the elastic biofilm. (**g**) The *x*-coordinate of center of mass versus time. The *x*-coordinate of the viscoelastic biofilm continues to increase in time as the dashpots in the Maxwell elements continue to increase in length. The *x*-coordinate of the elastic biofilm reaches equilibrium when the springs in the Stokes elements reach equilibrium. (**h**) The *y*-coordinate of center of mass versus time. Relative to the change in the *x*-coordinate, the change of the *y*-coordinate is an order of magnitude smaller, indicating the flow acts primarily to shear the biofilms.

We first test the response of the elastic and viscoelastic biofilms to shear flow at various values of $u_\text {max}$. A comparison of the deformation of the two biofilm structures is shown in Fig. 2 for values of $u_\text {max} = 1,\,3,\,5\times 10^{-6}$ m/s after 2.5 h in shear flow. The biofilm shapes indicate that the strain of the viscoelastic biofilm (Fig. 2d–f) exceeds that of the elastic one (Fig. 2a–c) and the difference in the strains of the two biofilms is greater at higher flow rates. Greater strains in viscoelastic materials are expected because they exhibit time-dependent stress-strain behavior and potentially undergo permanent deformation under stress. Additionally, at higher flow rates protrusions appear in the viscoelastic biofilm that are absent in the elastic biofilm^62^.

To quantify the extent of the biofilm deformation, we compute the *x*- and *y*-coordinates of the centers of mass, which are plotted versus time in Fig. 2g, h. After a few hundred seconds, the *x*-coordinate (Fig. 2g) of the elastic biofilm changes little as the Stokes elements are nearly in equilibrium with the fluid flow, while the *x*-coordinate of the viscoelastic biofilm moves downstream at a nearly constant speed as the dashpots in the Maxwell elements continually increase in length. Relative to the *x*-coordinate, the *y*-coordinate (Fig. 2h) of both biofilms changes little over 2.5 h. The change in the *y*-coordinate is about an order of magnitude smaller than the *x*-coordinate, which indicates the flow acts primarily to shear the biofilms.

**Figure 3:**
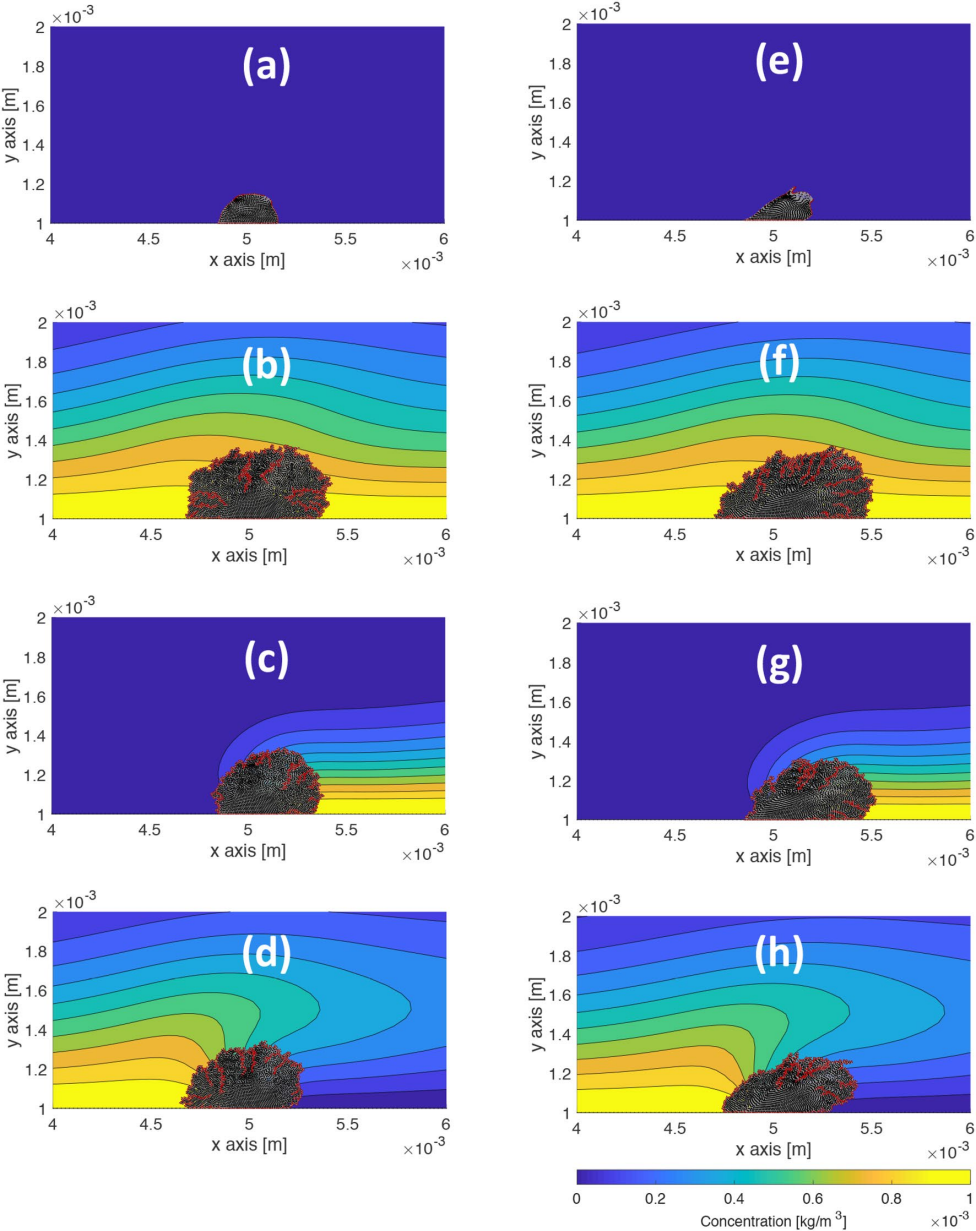
Comparison of growth and deformation in nutrient configurations and shear flow between an elastic biofilm ($E = 50$ Pa) and a viscoelastic biofilm ($E = 50$ Pa, $\eta = 5\times 10^4$ Pa s) after 2.5 h. The red lines indicate the boundary of the biofilm determined with the MATLAB function polyshape. (**a**)–(**h**) Top to bottom: no-concentration, full-stream concentration, downstream concentration, upstream concentration. (**a**)-(**d**) Elastic biofilm. (**e**)-(**h**) Viscoelastic biofilm. The viscoelastic biofilm grows further downstream than the elastic biofilm in each nutrient configuration.

Next, we test the growth of the elastic biofilm and the viscoelastic biofilm in the presence of the four nutrient configurations shown in Fig. 1b–e for values of $u_\text {max} = 0,\,1,\,2,\,3,\,4,\,5\times 10^{-6}$ m/s. The results of the simulation for the four concentration configurations under a flow value of $u_\text {max}=5\times 10^{-6}$ m/s at 2.5 h are shown in Fig. 3. In each case, the viscoelastic biofilm grows further downstream than the elastic one. Zoomed-in snapshots of the biofilm shapes in Fig. 3 are shown in Supplementary Information-4.

**Figure 4:**
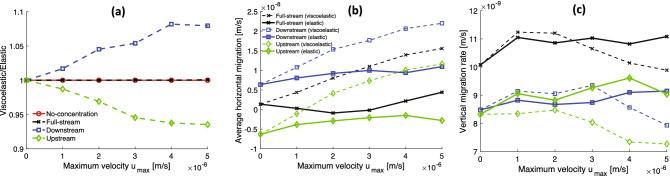
Comparison of net growth and migration in full-stream, downstream, and upstream nutrient configurations over a range of shear flow values between an elastic biofilm ($E = 50$ Pa) and a viscoelastic biofilm ($E = 50$ Pa, $\eta = 5\times 10^4$ Pa.s). (**a**) The ratio of the biomass of the viscoelastic biofilm to the biomass of the elastic biofilm at 2.5 h. The elastic biofilm tends to overestimate the growth in the upstream configuration and underestimate the growth in the downstream configuration. (**b**) The migration rate in the *x*-direction, i.e., the velocity of the *x*-coordinate of the center of mass. The nearly constant offset between the migration rate in graphs of the downstream and upstream configurations provides a measure of the migration rate toward the nutrient source. The full-stream configuration is approximately the average of the downstream and upstream migration rate, which provides a measure of the migration rate in response to shear flow. (**c**) The migration rate in the *y*-direction, i.e., the velocity of the *y*-coordinate of the center of mass. The elastic biofilm overestimates the vertical migration rate at large values of $u_\text {max}$.

The difference between the amount of growth of the elastic biofilm and the viscoelastic biofilm in our simulations is caused by the interplay between their growth toward the nutrient source and their downstream deformation in response to the shear flow. To quantify the difference in the net growth between the two biofilms, we take the ratio of their biomass at 2.5 h for each of the four nutrient configurations under each flow speed. The results, shown in Fig. 4a, indicate the simulations with the elastic biofilm overestimate the growth when the nutrient source is located upstream and underestimate the growth when the nutrient source is located downstream. This is an intuitive result because the shear flow tends to push the viscoelastic biofilm into the vicinity of the downstream nutrient source more than the elastic biofilm (Fig. 3c,g), while the elastic biofilm resists moving downstream and remains in the vicinity of the upstream nutrient source more than the viscoelastic biofilm (Fig. 3d,h). The greater growth of the elastic biofilm in the upstream configuration and the greater growth of the viscoelastic biofilm in the downstream configuration tend to cancel each other when the nutrient source is distributed symmetrically in the full-stream (Fig. 3b,f) and in the no-concentration case (Figs. 3a,e), giving a ratio near unity (Fig. 4a).

The growth and deformation of the biofilms results in the net migration of their centers of mass along the bottom of the channel. To measure the migration rate, we fit a least squares line to the *x*- coordinate of the center of mass versus time, shown in Fig. 4b, and a least squares line to the *y*-coordinate of the center of mass versus time, shown in Fig. 4c. In Supplementary Information-4 we show the linear fits over 2.5 h with the residuals displayed to verify their accuracy. The results for the *x*-direction migration rate (Fig. 4b) indicate the viscoelastic biofilm always migrates further downstream than the elastic biofilm. This result is intuitive because the viscoelastic biofilm always tends to deform more downstream in response to the fluid flow more than the elastic biofilm. In the case where the nutrient source is located upstream, the net migration of the biofilm can be in the upstream direction. At small flow speeds the viscoelastic biofilm migrates upstream, while at high flow speeds it migrates downstream. The transition from upstream to downstream migration in the upstream configuration does not occur for the elastic biofilm, as it migrates upstream at each flow rate. The migration rate of the two biofilms is also different in the full-stream nutrient configuration even though their net growth is approximately equal (Fig. 4a).

The fact that the *x*-direction migration rate downstream appears graphically as an approximately vertical shift from the *x*-direction migration rate upstream for both biofilms in 4b suggests that the migration rate due to growth and the migration rate due to shear deformation is approximately additive, and the migration rate due to growth is approximately constant. Assuming the upstream velocity is $U_\text {up} \approx -U_\text {growth} + U_\text {shear}$ and the downstream velocity is $U_\text {down} \approx U_\text {growth} + U_\text {shear}$, where $U_\text {growth}$ and $U_\text {shear}$ are the magnitude of the contribution to the migration rate due to growth and the shear flow respectively, then $U_\text {growth} \approx (1/2)(U_\text {down} - U_\text {up})$. Thus, taking the mean value of $U_\text {growth}$ over the range of $u_\text {max}$ for the viscoelastic and elastic biofilm gives a measure of for the migration rate due to growth: $U_\text {growth} = (0.021\pm 0.002)$ mm/hr where the reported error is the standard deviation of $U_\text {growth}$. The migration speed due to the shear flow, $U_\text {shear}$, can also be estimated from the data as $U_\text {shear} \approx (1/2)(U_\text {down} + U_\text {up}) \approx U_\text {full}$, where $U_\text {full}$ is the migration speed of the biofilm in the full-stream nutrient configuration. The migration speed due to the shear flow at $u_\text {max}=5\times 10^{-6}$ m/s is $U_\text {shear} \approx 0.056$ mm/h for the viscoelastic biofilm and $U_\text {shear} \approx 0.016$ mm/h for the elastic biofilm. The *y*-coordinate migration rate, shown in Fig. 4c, is similar for both biofilms at small flow rates, while at large flow rates, the elastic biofilm overestimates the *y*-direction migration rate.

### Viscoelastic biofilms

Measurements of biofilms reported in the literature indicate that over eight orders of magnitude of the elastic shear modulus ($10^{-2} - 10^{6}$ Pa) biofilms have a common viscoelastic relaxation time of about 18 min^10^. We use our model to compare the growth and deformation of viscoelastic biofilms with relaxation times near 18 min over a range of two orders of magnitude in elastic modulus: $10-1000$ Pa. The range was chosen to give reasonable numerical results for the values of shear flow used in our simulations, $u_\text {max} = 0,1,2,3,4,5\times 10^{-6}$ m/s over 2.5 hour-duration. For these flow rates, biofilms softer than 10 Pa have strains so large that numerical errors could become significant, while biofilms stiffer than 1000 Pa have strains so small that differences between biofilms became negligible. Moreover, large strains in our simulations are undesirable because biofilms are known to exhibit strain hardening at large strain values^43^, which our model does not capture. Nevertheless, the range of elastic moduli we simulate reliably capture a typical range of 70-700 Pa measured in microfluidic experiments^43^.

The relaxation time of the biofilm $\tau$ is related to the elastic modulus *E* and the axial viscosity $\tilde{\eta }_\text {axial}$ (i.e., the viscosity measured from axial stress versus axial strain) as

32 $$\begin{aligned} \tau = \frac{\tilde{\eta }_\text {axial}}{E}, \end{aligned}$$

where

33 $$\begin{aligned} \tilde{\eta }_\text {axial} = \eta \left( 1 + \frac{\eta _\text {water}}{2\eta }\right) . \end{aligned}$$

In the Supplementary Information-2, we derive equations Eqs. (32) and (33) and discuss the quantity $\eta _\text {water} = 1.02\times 10^3$ Pa.s, which is the drag coefficient of a fluid node in the simulation divided by the discretization length of the Lagrangian biofilm structure. In earlier studies^24,28^, the viscosity of the water alone was used to simulate the viscous response of biofilms without accounting for viscosity in the biofilm structure. We note that for a value of $\eta = 5\times 10^4$ Pa.s, $\eta _\text {water}$ accounts for a 10% correction to $\tilde{\eta }_\text {axial}$. Thus, for values of $E > 50$ Pa, the viscosity of the water contributes less than 10% percent to the dashpot viscosity $\eta$ determined by requiring $\tau = 18$ min.

**Figure 5:**
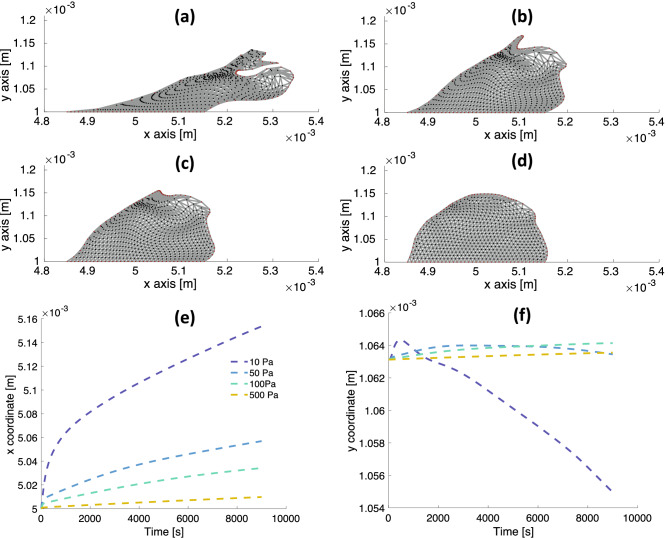
Comparison of deformation in shear flow between viscoelastic biofilms with relaxations time of 18 min at a flow rate of $u_\text {max} = 5\times 10^{-6}$ m/s. (**a**)-(**d**) From left to right, top to bottom: $E = 10,\,50,\,100,\,500$ Pa. In shear flow, soft biofilms deform more and develop larger protrusions than stiff biofilms. (**e**) The *x*-coordinate of center of mass versus time. All the biofilms have a relaxation time near 18 min, which requires the viscosity increase in proportion to the elastic modulus. Thus, the asymptotic slope approaches zero in the limit of large elastic modulus. (**f**) The *y*-coordinate of center of mass versus time. Relative to the change in the *x*-coordinate, the change of the *y*-coordinate is an order of magnitude smaller, indicating the flow acts primarily to shear the biofilms.

We test the deformation of the viscoelastic biofilms in the response to shear flow without nutrient present (Fig. 1b) for the range of elastic modulus values $E = 10,\, 50,\, 100,\, 500$ Pa, at a flow rate of $u_\text {max} = 5\times 10^{-6}$ m/s. A comparison of the deformation of the biofilms is shown in Fig. 5. The results indicate that softer viscoelastic biofilms undergo larger strains and develop larger protrusions than stiffer biofilms (Fig. 5a–d). For each biofilm, the *x*-coordinate of the center of mass moves in the direction of the fluid flow (Fig. 5e). The closer to zero the asymptotic slope of the *x*-coordinate versus time becomes, the larger the elastic modulus is. It is expected that viscoelastic biofilms with large values of *E* behave like rigid biofilms with near zero strain rates (Fig. 2g). Increasing *E* at a given shear stress reduces the strain of the biofilm (Eq. 7), and reducing the strain at fixed relaxation time ($\tau \sim \eta /E$ for large *E*) reduces the strain rate of the biofilm (Eq. 8). The change in the *y*-coordinate of the center of mass over 2.5 h is about an order of magnitude smaller then the *x*-coordinate of the center of mass for all the viscoelastic biofilms (Fig. 5f), which indicates the flow acts primarily to shear the biofilms.

**Figure 6:**
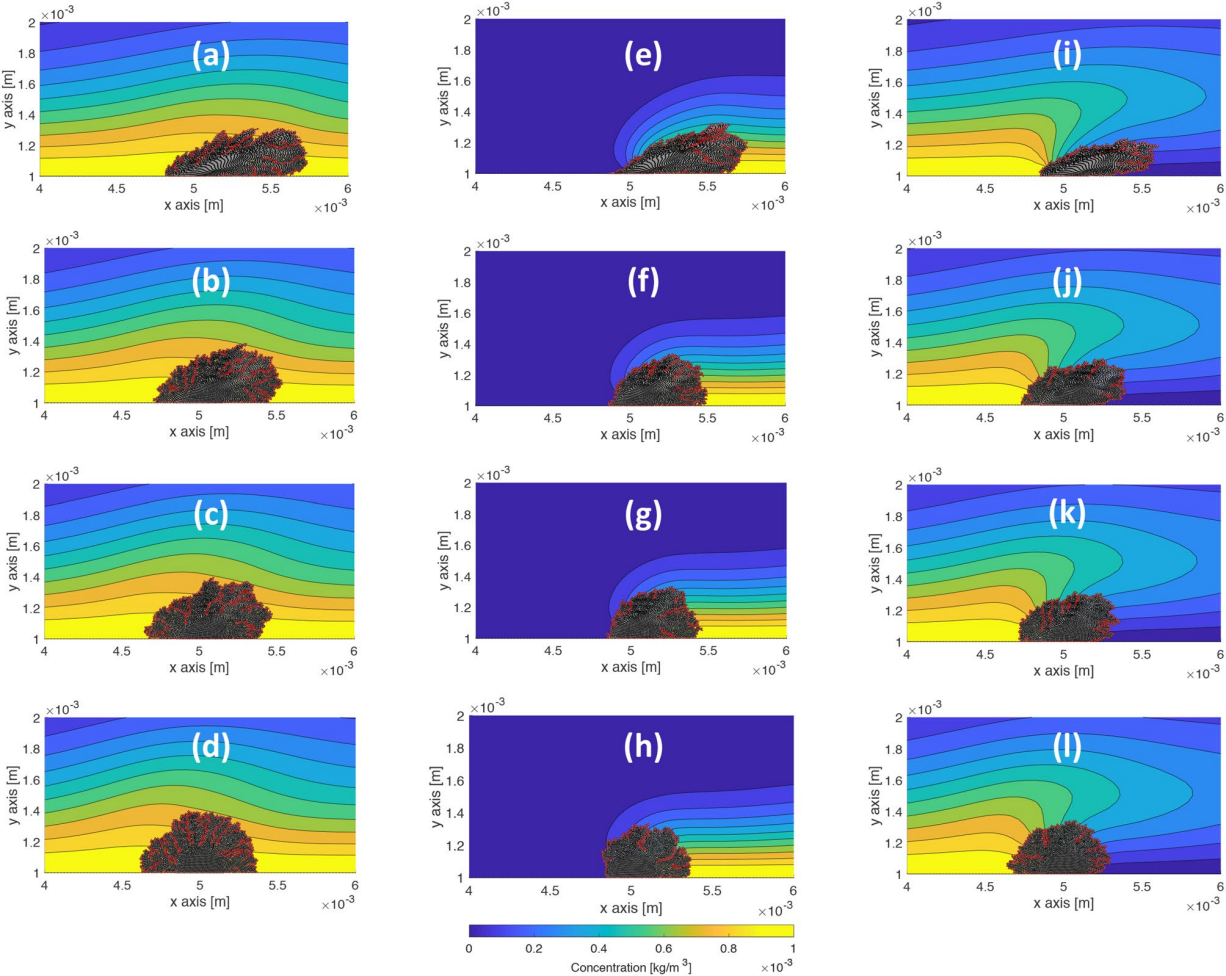
Comparison of growth and deformation of viscoelastic biofilms with three nutrient source configurations and at a shear flow value $u_\text {max} = 5\times 10^{-6}$ m/s. The red lines indicate the boundary of the biofilm determined with the MATLAB function polyshape. (**a**)-(**l**) Left to right: full-stream concentration, downstream concentration, upstream concentration; top to bottom: $E = 10,\,50,\,100,\,500$ Pa. Softer biofilms grow further downstream than the stiffer biofilms in each nutrient configuration.

Next, we simulate the growth and deformation of the viscoelastic biofilms in the presence of the three nutrient configurations shown in Fig. 1c–e for values of $E = 10,\, 50,\, 100,\, 500,\, 1000$ Pa at $u_\text {max} = 5\times 10^{-6}$ m/s. For other parameter values, see Table 1. Images of the results are shown in Fig. 6 for the full-stream, downstream, and upstream configurations. Figure 6a–d suggest all viscoelastic biofilms grow a similar amount in the full-stream nutrient configuration. The softest biofilm appears to grow more in the downstream configuration Fig. 6e as compared to the upstream configuration Fig. 6i, while the stiffest biofilm appears to grow more in the upstream configuration Fig. 6l as compared to the downstream configuration Fig. 6h. Zoomed-in snapshots of the biofilms in Fig. 6 are shown in Supplementary Fig. 3.

**Figure 7:**
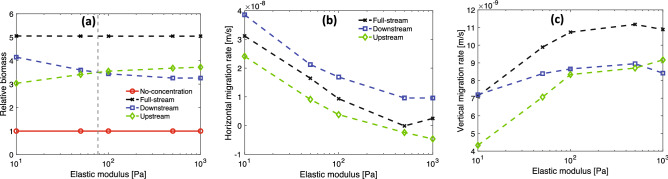
Comparison of growth and migration rate of viscoelastic biofilms in different nutrient source configurations and at a shear flow value $u_\text {max} = 5\times 10^{-6}$ m/s over a range of elastic moduli. (**a**) Ratio of the biomass of the biofilms at 2.5 h to their initial biomass. Below $E\approx 80$ Pa biofilms grow more in a downstream configuration relative to an upstream configuration. Above $E\approx 80$ Pa biofilms grow more in an upstream configuration relative to a downstream configuration. In the symmetric full-stream and no-concentration configurations, biofilm growth is independent of the elastic modulus. (**b**) Horizontal migration rate. The nearly constant offset between the downstream and upstream configurations provides a measure of the migration rate toward the nutrient source. The full-stream configuration provides a measure of the migration rate in response to shear flow. In the limit of large elastic modulus, the strain rate becomes negligible and the migration rate is due primarily to growth toward the nutrient source. (**c**) Vertical migration rate. Soft biofilms in the downstream and the full-stream configurations have similar migration rates. Stiff biofilms in downstream and upstream configurations have similar migration rates.

To compare how the nutrient configuration affects the growth of the different viscoelastic biofilms in shear flow, we compute the biomass of each biofilm at 2.5 h relative to the initial biomass for $u_\text {max} = 5\times 10^{-6}$ m/s. The results, shown in Fig. 7a, indicate that soft biofilms grow more in a downstream nutrient configuration while stiff biofilms grow more in an upstream configuration. Thus, there is a crossover, which occurs at $E \approx 80$ Pa, shown as the vertical dashed line in Fig. 7a. The crossover value of *E* is not universal but depends on the specific values of flow rate $u_\text {max}$ and the nutrient source concentration $c_0$. The greater growth of soft bioflims in the downstream case and the greater growth of the stiff biofilms in the upstream case tend to cancel when the nutrient is symmetrically distributed as in the full-stream and no-concentration configurations, which leads to equal net growth over the full range of elastic moduli.

As seen in Fig. 6, when the viscoelastic biofilms grow, they migrate horizontally along the bottom of the channel and vertically away from the wall. To measure the horizontal and vertical migration rates, we fit least squares lines to the *x*-coordinate of the center of mass versus time and the *y*-coordinate of the center of mass versus time respectively for each biofilm in each nutrient configuration. In Fig. 7b, the horizontal migration rate is plotted versus *E* in the downstream, full-stream, and upstream configurations. The results can be understood intuitively in terms of the downstream migration rate $U_\text {down} \approx U_\text {growth} + U_\text {shear}$, the upstream migration rate $U_\text {up} \approx -U_\text {growth} + U_\text {shear}$, and the full-stream migration rate $U_\text {full} \approx U_\text {shear}$ as discussed in the previous section. For all values of *E*, the difference between the downstream rate and the upstream rate gives a measure of the speed with which the biofilm migrates toward the nutrient, $U_\text {growth} \approx (1/2)(U_\text {down} - U_\text {up}) =(0.024\pm 0.002)$ mm/h, which agrees with the result in the last section obtained by averaging $U_\text {growth}$ over the range of $u_\text {max}$. At the elastic modulus value $E = 10$ Pa for the softest biofilm, $U_\text {shear} \approx 0.112$ mm/h, and at $E = 1000$ Pa for the stiffest biofilm, $U_\text {shear} \approx 0.009$ mm/h. In the limit of large values of *E*, the biofilm undergoes negligible strains due to the shear flow and thus $U_\text {shear} \rightarrow 0$ and the migration rate is dominated by $U_\text {growth}$: in the upstream configuration $U_\text {up} \rightarrow -U_\text {growth}$, in the downstream configuration $U_\text {down} \rightarrow U_\text {growth}$, and in the symmetric full-stream configuration $U_\text {full}\rightarrow 0$.

In Fig. 7c, the vertical migration rate is plotted versus *E* in the downstream, full-stream, and upstream nutrient configurations. For small values of *E*, the biofilm grows in the downstream configuration similarly to the full-stream configuration, while for large values of *E*, the biofilm grows in the downstream configuration similarly to the upstream configuration. These behaviors can be understood as follows: For small values of *E* the biofilm is strained most downstream by the shear flow. In the downstream configuration, this large deformation means the biofilm is surrounded by the nutrient concentration, thus its local environment is like that of the biofilm in the full-stream configuration. In the upstream configuration, the biofilm is pushed out of the region of highest concentration, thus it grows more slowly. For large values of *E*, the biofilm is barely strained by the shear flow, thus the biofilm in the upstream configuration and the biofilm in the downstream configuration are surrounded by similar amounts of nutrient.

## Discussion

In this work, we present a new model to simulate the growth of elastic and viscoelastic biofilms in shear flow under different nutrient source configurations. The interaction between the biofilm structure and the fluid is computed using the Immersed Boundary Method (IBM) and the growth is simulated using a probabilistic rule based on local nutrient concentrations. Simulations are completed using the open source IB2d software^44–46^, which we modified to accommodate a reaction term in the advection-diffusion equation solver and to implement the growth scheme. The viscoelastic and chemical kinetic parameters in the simulations are taken directly from experimental values reported in the literature (Table 1).

Our method of applying IBM resolves the challenge^25^ of determining viscoelastic parameter values a priori rather than having to perform a parametric search to match simulations to experiments. In IBM, the point forces exerted on the nodes of the Lagrangian structure must be transformed into a force density function. Typically this is accomplished (for an elastic structure) by defining “stiffness constants” that have dimensions of force per generalized Lagrangian volume. The difficulty is that these constants do not have a clear interpretation in terms of the actual spring constants or the elastic moduli. In a square lattice, elementary theory gives the interatomic spring constant *k* in terms of the elastic modulus *E* and the interatomic spacing *d* as $k=Ed$, independently of the dimensionality of the lattice^63^. Thus, it should be possible, given a Lagrangian structure with spacing *d* and an elastic modulus *E*, to a priori determine *k*. To accomplish this, we define the spring force between nodes using the spring constant $k=Ed$ and use Dirac delta functions to define force densities directly from the point forces acting on the nodes of the Lagrangian structure. This procedure enables an exact evaluation of the force spreading equation (Eq. 3), which results in body forces in the Eulerian domain that take the same form as those employed in other computational fluid dynamics methods (Eq. 14), for example in the Method of Regularized Stokeslets^64^. The technical details of our approach are given in Supplementary Information-1 and -2, and the resulting simulation is validated in Supplementary Information-3.

Many earlier models treated biofilms as purely elastic structures, though biofilms are known to be viscoelastic. Thus, we use our model to find differences between simulations of a purely elastic biofilm and of a viscoelastic biofilm with the same elastic modulus of 50 Pa. In simulations with shear flow but without a nutrient source, the viscoelastic biofilm develops a morphological structure with protrusions that the purely elastic biofilm does not develop. In simulations with shear flow and an asymmetric nutrient source, the elastic biofilm underestimates or overestimates growth rates depending on the configuration of the nutrient supply in the channel. With the nutrient supply located upstream from the biofilm, the elastic biofilm overestimates the growth rate. With the nutrient supply located downstream, the elastic biofilm underestimates the growth rate. In all nutrient configurations, even in the symmetric full-stream and no-concentration configurations, in which the growth rates are equal, the viscoelastic biofilm migrates more downstream than the elastic biofilm. In general, the differences between the elastic and viscoelastic biofilms are larger for higher flow rates. These results should be useful for future efforts to model biofilms using immersed structures because they illustrate some limitations of using a purely elastic structure.

Our simulations capture the biological reality that biofilms possess a common viscoelastic relaxation time of about 18 min over a wide range of elastic moduli^10^. We found that an interplay between biofilm deformation due to shear flow and biofilm growth toward a nutrient source resulted in a crossover value of the elastic modulus. Biofilms with elastic moduli below the crossover value grew faster when the nutrient source was located downstream than they did when the nutrient source was located upstream. Conversely, biofilms with elastic moduli above the crossover value grew faster when the nutrient source was located upstream than they did when the nutrient source was located downstream. We also found that biofilms migrated along the bottom of the channel wall. Soft biofilms migrated downstream in all nutrient configurations but migrated faster when the nutrient source was located downstream. Stiff biofilms migrated downstream when the nutrient source was located downstream but migrated upstream when the nutrient source was located upstream. Our results agree with the biophysical principle that by tuning their viscoelastic parameters, perhaps by modulating their polysaccharide production^7,16,18^, biofilms can adapt to their local nutrient conditions in shear flow to maximize their growth rate and their migration rate toward a nutrient source. In this work, we focus on the interplay between biofilm growth and deformation over a few hours. In the future, to further capture real characteristics and evolution of a viscoelastic biofilm, we will extend our model to include spatial porosity and heterogeneous structure and to exhibit detachment phenomena.

## Supplementary information

Supplementary material 1.

Supplementary material 2.

Supplementary material 3.

Supplementary material 4.

Supplementary material 5.

Supplementary material 6.

Supplementary material 7.

Supplementary material 8.

Supplementary material 9.

Supplementary material 10.

Supplementary material 11.

Supplementary material 12.

Supplementary material 13.

Supplementary material 14.

## Data Availability

Our software package is available at Code Ocean (https://codeocean.com/) for: *Creep test*: https://doi.org/10.24433/CO.9059597.v1. *Simulation of biofilm growth and deformation*: https://doi.org/10.24433/CO.0816145.v1.
