# Supplementary material for: Biofilm viscoelasticity and nutrient source location control biofilm growth rate, migration rate, and morphology in shear flow
Source: Sci Rep. 2021 Aug 9;11:16118. doi: 10.1038/s41598-021-95542-1 (PMC8352988; doi:10.1038/s41598-021-95542-1)
Supplement: Supplementary file 13 — Supplementary material 13. [file 41598_2021_95542_MOESM13_ESM.pdf]

Biofilm Viscoelasticity and Nutrient Source Location Control Biofilm Growth Rate, Migration Rate, and Morphology in Shear Flow

Hoa Nguyen[1], Abraham Ybarra[1], Hakan Başağaoğlu[2], and Orrin Shindell[3,*]

[1]Department of Mathematics, Trinity University, San Antonio, TX 78212, USA
[2]Evolution Online LLC, San Antonio, TX 78292, USA
[3,*]Department of Physics & Astronomy, Trinity University, San Antonio, TX 78212, USA
[*]oshindel@trinity.edu

We describe our method of defining the 'force density function' on the Lagrangian structure in the Immersed Boundary Method in Supplementary Information-1. Then in Supplementary Information-2 we show how to determine the viscoelastic parameters for the force elements in our Lagrangian structure directly from experimental measurements. The results from our simulations of the creep tests match the predictions from our calculations of the viscoelastic parameters and are shown in Supplementary Information-3. To demonstrate how the concentration location, shear flow, and viscoelastic properties affect the biofilm morphology, zoomed-in snapshots of Fig. 3 and Fig. 6 in the main text are displayed in Supplementary Information-4. The details of the linear least squares fits to compute the migration rates in Fig. 4 and Fig. 7 in the main text are presented in Supplementary Information-5. Finally, Supplementary Information-6 will be updated to include a link to our code after our manuscript is accepted.

Supplementary Information-1: Force Density in the Immersed Boundary Method

Here we present the logic of our approach to the Immersed Boundary Method (IBM) that allows for an *a priori* determination of simulation parameters from experimental data. The essential problem the IBM solves is this: Given a structure with internal stresses immersed in a fluid, determine the resulting fluid flow and motion of the structure. The particles of the immersed structure are treated as equivalent to fluid particles, thus the density of forces acting on the structure may be thought of as body forces acting on fluid particles. From a computational standpoint, however, the Eulerian coordinates of the fluid and the Lagrangian coordinates of the structure are not in general coincident. Thus, the force density on the Lagrangian structure must be transformed into body forces on the fluid in the Eulerian domain before they may be input into the Navier-Stokes equations. Moreover, it may be convenient to define the Lagrangian structure with a lower dimensionality than the fluid. The transformation of force density to an Eulerian domain from a Lagrangian domain of equal or lower dimension is accomplished with the integral transform equation

$$\mathbf{f}(\mathbf{x}) = \int_{\mathscr{L}} \mathbf{F}(\mathbf{X})\delta(\mathbf{x}-\mathbf{X})d\mathscr{L}, \tag{1}$$

where $\mathbf{f}$ is the body force acting on fluid particles defined in Eulerian coordinates $\mathbf{x}$, $\mathbf{F}$ is the force density acting on the immersed structure defined in Lagrangian coordinates $\mathbf{X}$, and $\mathscr{L}$ is the Lagrangian domain with $d\mathscr{L}$ the generalized volume element of $\mathscr{L}$. The kernal evaluated at a particular Lagrangian point $\mathbf{X}_0$ is the product of Dirac delta functions written in Cartesian coordinates $(x_1, \cdots, x_n)$ as

$$\delta(\mathbf{x}-\mathbf{X}_0) \equiv \prod_{i=1}^{n} \delta(x_i - X_{i0}), \tag{2}$$

where n is the dimension of the Eulerian space. We also note that the dimensions of $\delta(\mathbf{x}-\mathbf{X})$ are such that $\delta(\mathbf{x}-\mathbf{X})d\mathscr{E}$ is dimensionless, where $\mathscr{E}$ is the Eulerian domain and $d\mathscr{E}$ its generalized volume element. Generally, Eq. 1 is computed by introducing a regularized delta function $\phi(\mathbf{x})$ and then performing a Riemann sum. In applications like ours that define the

immersed structure as a network of elastic or viscoelastic elements it is not necessary to perform the Riemann sum nor is it necessary to define stiffness constants with units of force per Lagrangian volume (or similar viscosity constants), which require a parametric search to fit to experimental data. Indeed, there is a connection between the Riemann sum and the stiffness constants; as we show below, the stiffness constant is the typical spring constant divided by the Lagrangian volume element.

To illustrate our method of defining a force density function on the Lagrangian structure and then evaluating Eq. 1, we use a simple intuitive analogy using mass and mass density. Consider a long thin uniform rod with mass M and length L. Define an"IBM" setup where the rod is represented as a one-dimensional Lagrangian fiber "immersed" in a two-dimensional Eulerian domain and the goal is to compute the mass of the rod by numerical integration in the Eulerian domain. Letting the rod lie along the x-axis from $-L/2$ to $L/2$ gives a Lagrangian domain $\mathbf{X}(x) = (x,0)$ with $x \in [-L/2, L/2]$, which may be discretized into a set of n points: $\mathbf{X}_k = (x_k, 0)$ where $x_k = -L/2 + (k)L/n$ for $k = 1, 2, \cdots, n$. The Eulerian domain has discretized Cartesian coordinates $\mathbf{x}_{ij} = (x_i, y_j)$ with grid widths Δx and Δy. Analagous to Eq. 1, the integral transform used to transform a mass density $\Lambda(\mathbf{X})$ defined on the Lagrangian fiber into the mass density $\lambda(\mathbf{x})$ in the Eulerian domain is,

$$\lambda(\mathbf{x}) = \int_{\mathscr{L}} \Lambda(\mathbf{X}) \delta(\mathbf{x} - \mathbf{X}) d\mathscr{L}. \tag{3}$$

Each point $\mathbf{X}_k$ has a mass $M_k = M/n$. A Dirac delta function may be used to define a mass density for the kth mass,

$$\Lambda_k(\mathbf{X}) = M_k \delta(x - x_k) \tag{4}$$

giving the mass density of the whole structure as

$$\Lambda(\mathbf{X}) = \sum_k \Lambda_k(\mathbf{X}) = \sum_k M_k \delta(x - x_k). \tag{5}$$

Inserting Eq. 5 into Eq. 3 gives

$$\lambda(\mathbf{x}) = \int_{-\frac{L}{2}}^{\frac{L}{2}} \left(\sum_k M_k \delta(x' - x_k) \right) \delta(\mathbf{x} - \mathbf{X}(x')) dx' = \sum_k M_k \delta\left(\mathbf{x} - \mathbf{X}_k\right) \tag{6}$$

where the delta function identity

$$\int_{x_k - \varepsilon}^{x_k + \varepsilon} f(x') \delta(x' - x_k) dx' = f(x_k) \tag{7}$$

has been employed with $\varepsilon \in \mathbb{R}^+$ and $f(x') = \delta(\mathbf{x} - \mathbf{X}(x'))$. Eq. 6 results in a mass density that takes the same form in Eulerian coordinates as it does in Lagrangian coordinates. Now the mass may be found by regularizing the delta function in Eq. 6: $\delta(x) \to \phi(x)$, and numerically integrating $\lambda(\mathbf{x})$ over the Eulerian domain,

$$M = \int_{\mathscr{E}} \lambda(\mathbf{x}) d\mathscr{E} \approx \sum_i \sum_j \left(\sum_k M_k \phi(\mathbf{x}_{ij} - \mathbf{X}_k) \right) \Delta x \Delta y \approx \sum_k M_k, \tag{8}$$

as expected.

A second way to define a mass density on the Lagrangian fiber, which is analogous to the standard way of defining force density with a stiffness constant, is to introduce a "mass constant" $\overline{M}_k$ of unknown value with units of mass density. The mass constant is defined by evaluating the Lagrangian mass density $\overline{\Lambda}(\mathbf{X})$ at $\mathbf{X}_k$:

$$\overline{\Lambda}(\mathbf{X}_k) \equiv \overline{M}_k \tag{9}$$

Spreading the mass density $\overline{\Lambda}(\mathbf{X})$ to the Eulerian grid using Eq. 3 gives

$$\overline{\lambda}(\mathbf{x}) = \int_{-\frac{L}{2}}^{\frac{L}{2}} \overline{\Lambda}(\mathbf{X}) \delta\left(\mathbf{x} - \mathbf{X}(x')\right) dx' \approx \sum_k \overline{M}_k \phi\left(\mathbf{x}_{ij} - \mathbf{X}_k\right) \Delta x' \tag{10}$$

where the delta function in Eq. 10 has been regularized: $\delta(\mathbf{x}) \to \phi(\mathbf{x})$, and the integral is written as a Riemann sum with $\Delta x' = L/n$. Numerically integrating $\overline{\lambda}(\mathbf{x})$ over the Eulerian domain gives

$$M = \int_{\mathscr{E}} \overline{\lambda}(\mathbf{x}) d\mathscr{E} \approx \sum_i \sum_j \left(\sum_k \overline{M}_k \phi\left(\mathbf{x}_{ij} - \mathbf{X}_k\right) \Delta x' \right) \Delta x \Delta y \approx \sum_k \overline{M}_k \Delta x'. \tag{11}$$

Comparing Eq. 8 and Eq. 11 shows that defining

$$\overline{M}_k = \frac{M_k}{\Delta x'} \tag{12}$$

gives the same result to the numerical calculation of the mass. The meaning of the mass constant is thus clear: It is the mass density defined by dividing the point mass attributed to $\mathbf{X}_k$ by the discretization size of the Lagrangian structure. The foregoing analysis with mass is equally valid for forces.

Supplementary Information-2: Viscoelastic Parameters

The biofilm in our simulation is constructed as a network of nodes connected by Stokes springs or Maxwell elements. The elastic modulus and viscosity of the biofilm may be determined by specifying the spring and dashpot constants. Rheology on biofilms shows that a typical shear modulus ranges from 50 Pa to 150 Pa and that a typical relaxation time is about 18 minutes[1]. In this section, we show how to determine the spring and dashpot constants *a priori* using these experimental data.

The elastic modulus (or Young's modulus) E is a material property that equals the ratio of axial stress to axial strain. It is an intensive property, i.e., it does not depend on the amount material. Thus, its value may be deduced from a single elastic element that comprises the material. Consider a material composed of a square lattice of nodes connected by Stokes elements. A single Stokes element with spring constant k is shown in Supplementary Fig. 1(a).

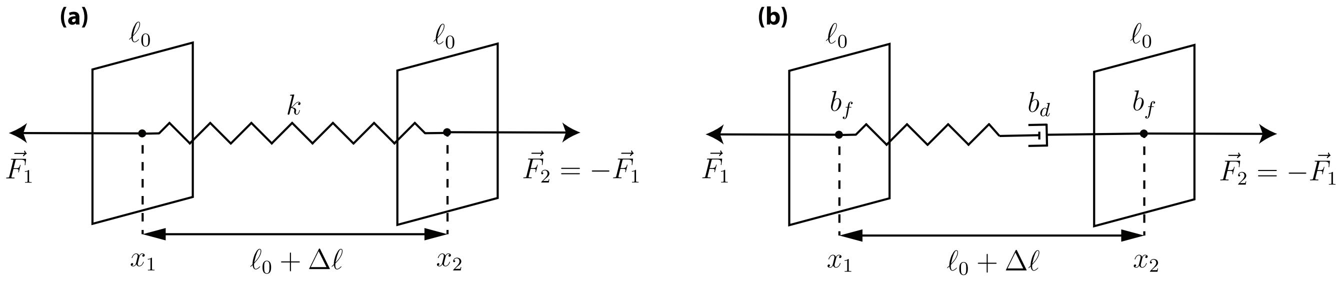

Supplementary Figure 1. (**a**) A single Stokes element; (**b**) A single Maxwell element

The resting length of the Stokes element is ℓ_0, and thus the cross-sectional area is ℓ_0^2. A tensile force of magnitude $F = \|\vec{F}_1\| = \|\vec{F}_2\|$ is applied to both sides of the element, which stretches the element by a distance $\Delta\ell$. At equilibrium

$$F = k\Delta\ell \Rightarrow \sigma_{\text{axial}} = \left(\frac{k}{\ell_0}\right)\gamma_{\text{axial}}, \tag{13}$$

where $\sigma_{\text{axial}} = F/\ell_0^2$ is the axial stress and $\gamma_{\text{axial}} = \Delta\ell/\ell_0$ is the axial strain. Thus, the elastic modulus is related to the Stokes element as

$$E = \frac{k}{\ell_0}. \tag{14}$$

The axial viscosity $\tilde{\eta}_{\text{axial}}$ is a material property that equals the ratio of the axial stress to the asymptotic axial strain rate. It too is an intensive property and thus may be deduced from a single viscoelastic element. Consider now a material composed of a square lattice of nodes connected by Maxwell elements. In the Maxwell element shown in Figure 1(b), the dashpot has a drag coefficient b_d, and there is fluid drag on the nodes with coefficient b_f. A tensile force of magnitude F is applied to both sides of the element. In the asymptotic regime, the length of the spring no longer changes, while the length of the element increases at a constant rate. In a non-inertial system, the forces must vanish; on left node x_1

$$0 = -F - b_f\dot{x}_1 + b_d(\dot{x}_2 - \dot{x}_1) \tag{15}$$

while on the right node x_2

$$0 = F - b_f\dot{x}_2 - b_d(\dot{x}_2 - \dot{x}_1). \tag{16}$$

Eq. 15 and Eq. 16 may be combined to give

$$F = b_d \left(1 + \frac{b_f}{2b_d}\right)(\dot{x}_2 - \dot{x}_1) \Rightarrow \sigma_{\text{axial}} = \frac{b_d}{\ell_0}\left(1 + \frac{b_f}{2b_d}\right)\dot{\gamma}_{\text{axial}}. \tag{17}$$

Thus, the axial viscosity is related to the parameters of the Maxwell element as

$$\tilde{\eta}_{\text{axial}} = \frac{b_d}{\ell_0}\left(1 + \frac{b_f}{2b_d}\right) = \eta\left(1 + \frac{\eta_{\text{f}}}{2\eta}\right). \tag{18}$$

where $\eta = b_d/\ell_0$ is the dashpot viscosity in Eq. 8 of the main text, and $\eta_{\text{f}} = b_f/\ell_0$ is defined analogously to η. The quantity b_f is determined by simulating the motion of a single node with a constant applied force. For an isolated node located a distance from the wall equal to the height of our biofilm with a force acting on it parallel to the wall, we find $b_f \approx 8.5 \times 10^{-3}$ Pa.m.s. The dynamic viscosity and density of the fluid in our simulation are listed in the Supplementary Table 1.

The biofilm rheology experiments[1] we aim to match with our simulations use a shear stress to measure the shear modulus G and shear viscosity $\tilde{\eta}$. Those experiments indicated the relaxation time of biofilms $\tau = \tilde{\eta}/G$ is roughly constant over a wide range of shear modulus and viscosity values. Generally $G = 3E$ for a material with Poisson ratio $1/2$. As shown in the next section, we find numerically $G \approx 3E$, where is E is calculated by Eq. 14. The shear viscosity obeys the same relationship, $\tilde{\eta} \approx 3\tilde{\eta}_{\text{axial}}$, with $\tilde{\eta}$ calculated by Eq. 18. It is expected that $G/E \approx \tilde{\eta}/\tilde{\eta}_{\text{axial}}$ because the shear strain rate deviates from the axial strain rate by the same fraction that the shear strain deviates from the axial strain. Thus, the relaxation time may be calculated directly from E and $\tilde{\eta}_{\text{axial}}$

$$\tau = \frac{\tilde{\eta}_{\text{axial}}}{E} \tag{19}$$

The spring constant k and dashpot constant b_d of Stokes and Maxwell elements connecting the nodes may be determined as follows:

1. Let ℓ_0 be the average length of the edges in the initial mesh.
2. Choose a value of G from an experiment to compute $E = G/3$. Then calculate $k = E\ell_0$.
3. Choose a value of τ from an experiment to compute $\tilde{\eta}_{\text{axial}} = \tau E$. Then calculate $b_d = \tilde{\eta}_{\text{axial}}\ell_0 - b_f/2$.

The dashpot viscosity can then be computed as $\eta = b_d/\ell_0$. In the next section, we present the results from a numerical creep test to show that choosing k and b_d in this fashion yields a simulated relaxation time that matches well with the chosen value of τ.

Supplementary Information-3: Creep Test

The creep test is conducted to determine the material properties of our biofilm model in a viscous fluid as it deforms under shear stress. Initially, the biofilm structure in Fig. 2(a) is discretized by DistMesh[2] to generate a uniform Delaunay triangulation. The edges represent the springs with the average lengths ℓ_0 to connect the nodes. Under a constant shear stress σ applied to the top nodes parallel to the wall, the biofilm structure deforms. Notice that the nodes at the bottom of the biofilm are always tethered to the bottom wall of the channel so the biofilm structure cannot be pulled away with the shear stress. Once the stress is released, the springs either go back to their initial resting lengths (for Stokes springs) or permanently deformed (for Maxwell elements). The creep function is defined as average strain of the top nodes and computed by[3]

$$\varepsilon(t) = \frac{1}{PN}\sum_{i=1}^{N} ||\mathbf{X}_i(t) - \mathbf{X}_i(0)||, \tag{20}$$

where P is the height of the biofilm and N is the total number of nodes on the top. $\mathbf{X}_i(t)$ is the position of the Lagrangian node at time t. The compliance function in[1] is the creep function divided by the shear stress.

First, we perform the creep test on a purely elastic biofilm structure (Supplementary Fig. 2(a)) comprising Stokes springs with elastic modulus $E = 50$ Pa. The shear stress σ is applied at every node at the top of the biofilm structure during the first 24,000 seconds. As shown in Supplementary Fig. 2(b), the compliance curve approaches an equilibrium before the stress is

turned off. The Stokes springs will eventually return to their initial configuration. This allows us to approximate the shear modulus G as the inverse of the equilibrium value of the compliance ($G \approx 2.7805E$).

Next, we perform the creep test again on the same biofilm structure comprising Maxwell elements. Our goal is to numerically find an appropriate value for the shear viscosity $\tilde{\eta}$ such that the biofilm structure has a similar behavior as the one for *S. mutans* biofilm presented Figure 2 of Shaw *et al.*[1] and the relaxation time $\tau = \tilde{\eta}/G$ is about 18 minutes. Supplementary Fig. 2(c) is shown with the dashpot viscosity $\eta = 50,000$ Pa.s where the strain rate $\dot{\gamma}$ is the slope of the creep curve (or the compliance curve $\times$ applied stress) right before the stress is released at the 10-minute mark. Due to a dashpot in series in the Maxwell elements and the fluid viscosity acting as a dashpot in parallel with the biofilm structure, the springs do not return to their initial resting lengths. The shear viscosity $\tilde{\eta}$ is calculated as $\sigma/\dot{\gamma}$ which is about $3.0554\tilde{\eta}_{\text{axial}}$. Hence, the relaxation time τ for our visco-elastic biofilm structure is computed as $\tilde{\eta}/G$ which equals 18.501 minutes. This agrees with the a-priori calculation $\tilde{\eta}_{\text{axial}}/E$ in the previous section to within 10% and the result presented by Shaw *et al.*[1]: The relaxation times of different bacterial biofilms are about 18 minutes. See Supplementary Table 1 for all the parameters used in our creep tests.

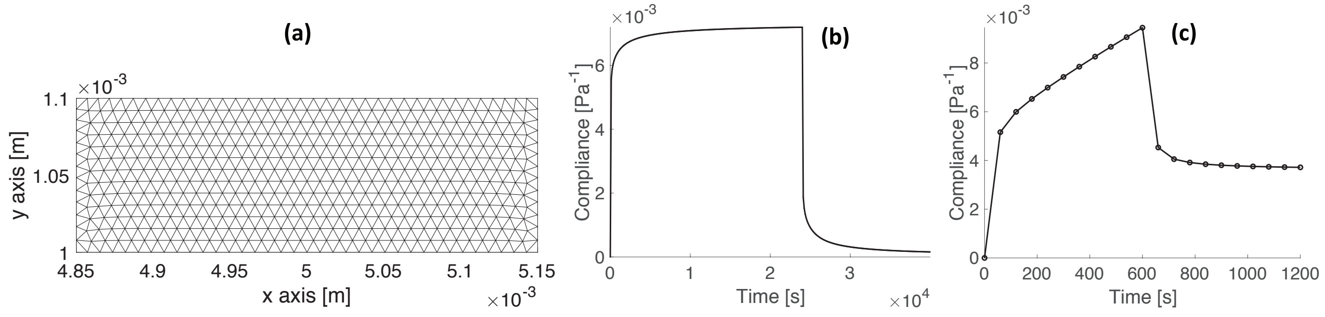

Supplementary Figure 2. (a) Initial biofilm structure for the creep test; (b) Creep test on an elastic structure; (c) Creep test on a viscoelastic structure

Supplementary Table 1. Parameters used the creep tests

Parameter	Symbol	Value	Unit	
Dynamic viscosity of the fluid	μ	9.31×10^{-4}	Pa.s	
Density of the fluid	ρ	1000	kg/m^3	
Length of channel	Lx	1×10^{-2}	m	
Width of channel	Ly	3×10^{-3}	m	
Eulerian grid size	$dx = dy = h$	4.17×10^{-5}	m	
Grid size on biofilm	$\ell_0 = ds$	$dx/5$	m	
Time step	δt	1×10^{-3}	s	
Applied stress	σ	50	Pa	
End time to apply stress	τ	2.4×10^{4}	s	Fig. 2(b)
		6×10^{2}	s	Fig. 2(c)
Elastic modulus of biofilm	E	50	Pa	
Dashpot viscosity	η	∞	Pa.s	Fig. 2(b)
Dashpot viscosity	η	5×10^{4}	Pa.s	Fig. 2(c)
Shear modulus	G	139.03	Pa	
Strain rate	$\dot{\gamma}$	3.24×10^{-4}	s^{-1}	

Supplementary Information-4: Comparison of Biofilm Growth, Migration, and Morphology

To compare the biofilm growth, migration, and morphology in different concentration configurations under a shear flow of $u_{\max} = 5 \times 10^{-6}$ m/s at 2.5 hours, zoomed-in snapshots of Fig. 3 and Fig. 6 in the main text are shown in the Supplementary Figures 3 and 4.

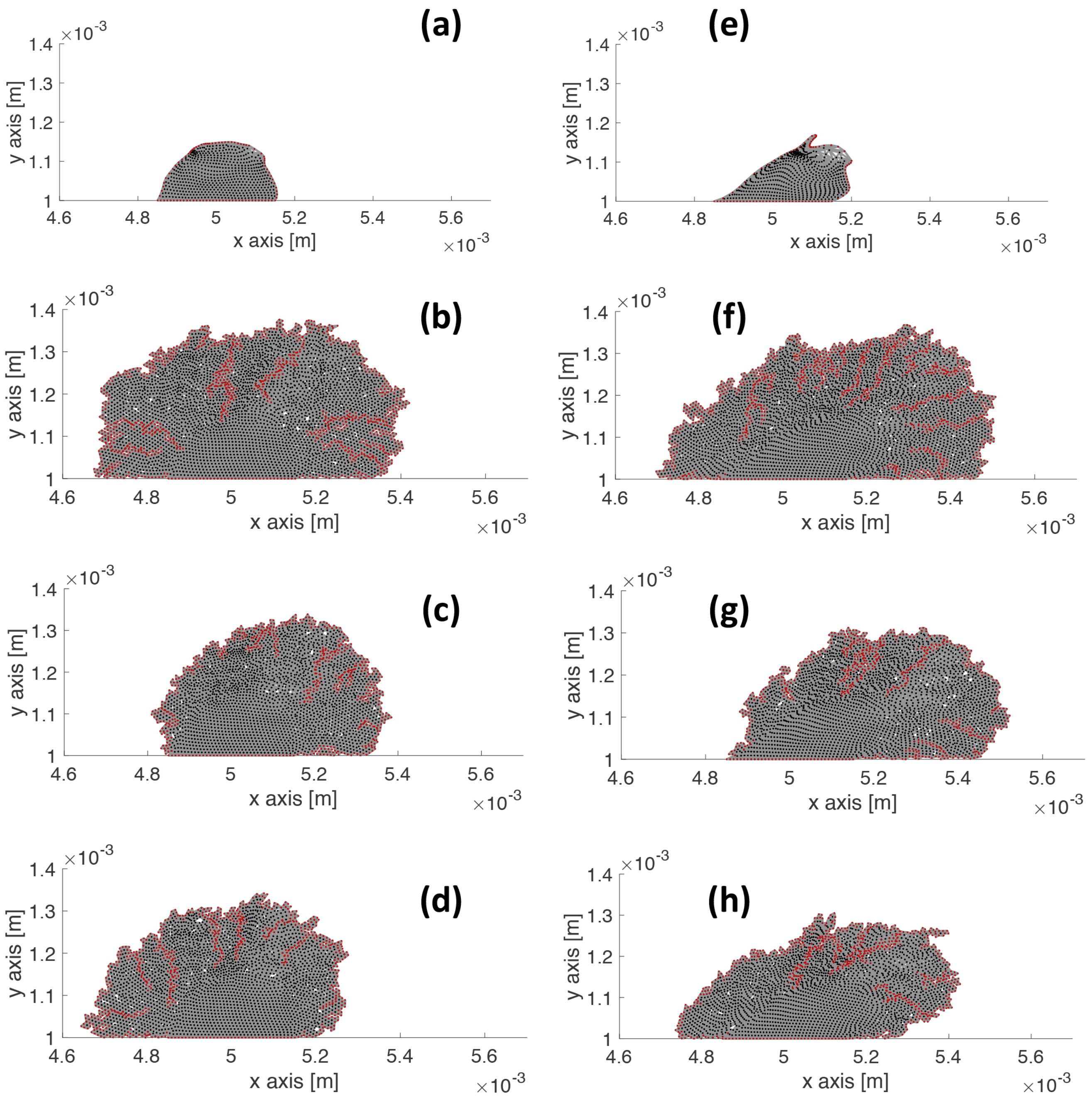

Supplementary Figure 3. While the elastic biofilm structures (left) grow and deform more locally around the initial shape, the viscoelastic biofilm structures (right) tend to lean in the flow direction in all four nutrient configurations (top to bottom: no-concentration, full-stream, downstream, and upstream).

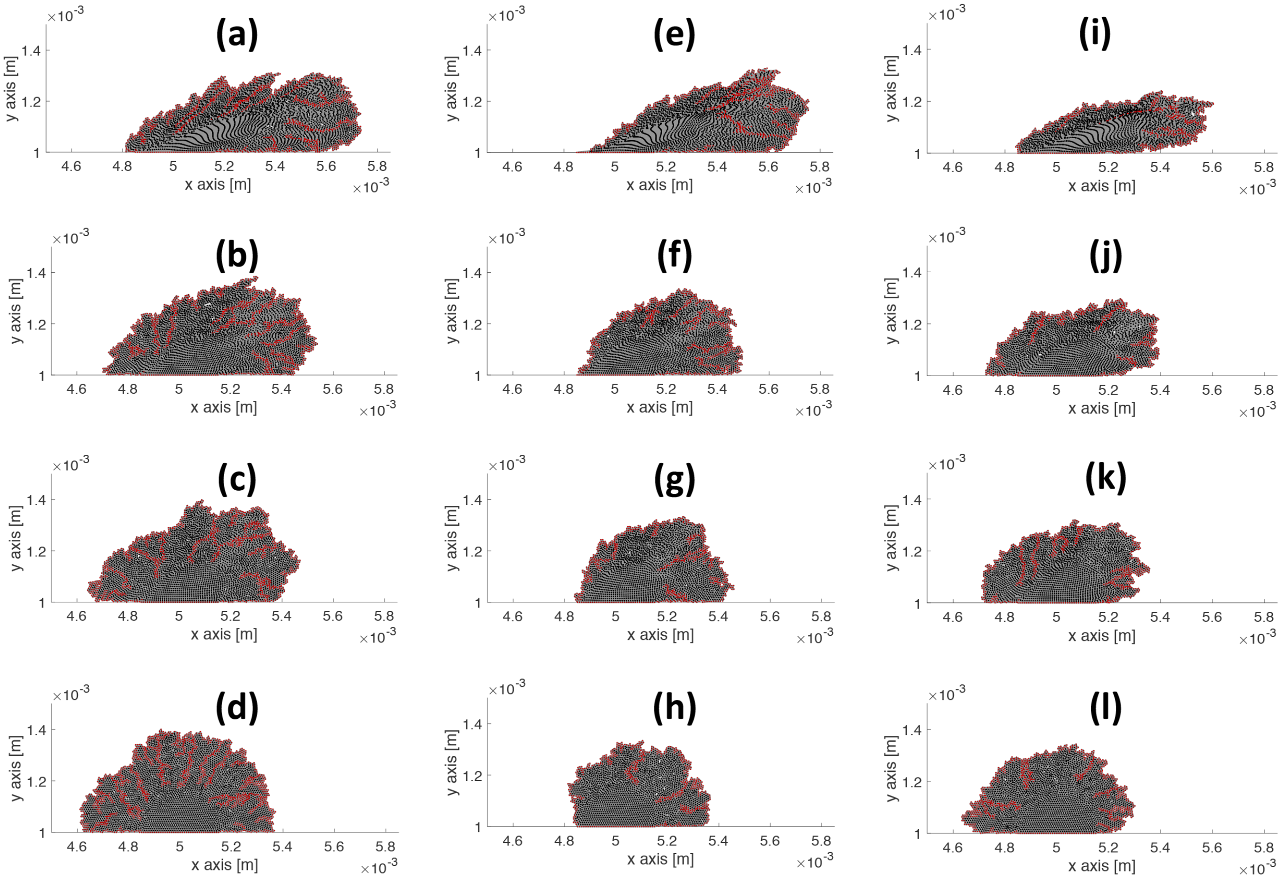

Supplementary Figure 4. Among the four viscoelastic biofilm structures (top to bottom: $E = 10, 50, 100, 500$ Pa) with the same relaxation time of 18 minutes, the softest biofilm (top) grows and deforms most in the shear flow direction for all three nutrient configurations (left to right: full-stream, downstream, and upstream). The stiffest one (bottom) maintains its growth and deformation locally around the initial shape. In comparison with the Supplementary Figure 3, the stiffest one behaves more like an elastic structure. This suggests real biofilm structures might change their rigidity to be more elastic or viscoelastic to control their growth, migration, and morphology as they interact with the surrounding fluid flow and nutrient sources.

Supplementary Information-5: Linear Least Squares Fits to Compute Migration Rates

To demonstrate how we find the horizontal and vertical migration rates in Fig. 4 and Fig. 7 of the main text, we present our linear least squares fits to our simulated data of the coordinates of the center of mass within 2.5 hours for three nutrient configurations (full-stream, downstream, and upstream). The slopes of these least squares lines are the migration rates. The L^2 norms of the residuals are also displayed for comparison. The Supplementary Figure 5 shows the fits related to the horizontal migration rates in Fig. 4(b) of the main text while the Supplementary Figure 6 is for the vertical migration rates in Fig. 4(c). Similarly, the Supplementary Figure 7 is for Fig. 6(b) of the main text while the Supplementary Figure 8 is for Fig. 6(c).

Supplementary Information-6: Available Code

Our software package is available at Code Ocean (https://codeocean.com/) for:
Creep test: https://doi.org/10.24433/CO.9059597.v1
Simulation of biofilm growth and deformation: https://doi.org/10.24433/CO.0816145.v1

Acknowledgements

This research was supported by NSF DMS-1720323 to HN, HB, and AY and NSF MRI-1531594 to HN. We thank Trinity University for the Summer Research Grant to OS and the Mach Fellowship to AY, and the provision of computational resources.

References

1. Shaw, T., Winston, M., Rupp, C., Klapper, I. & Stoodley, P. Commonality of elastic relaxation times in biofilms. *Phys. Rev. Lett.* **93**, 098102 (2004).

2. Persson, P.-O. & G.Strang. A simple mesh generator in MATLAB. *SIAM Rev.* **46**, 329–345 (2004).

3. Wróbel, J., Lynch, S., Barrett, A., Fauci, L. & Cortez, R. Enhanced flagellar swimming through a compliant viscoelastic network in Stokes flow. *J Fluid Mech* **792**, 775–797 (2016).

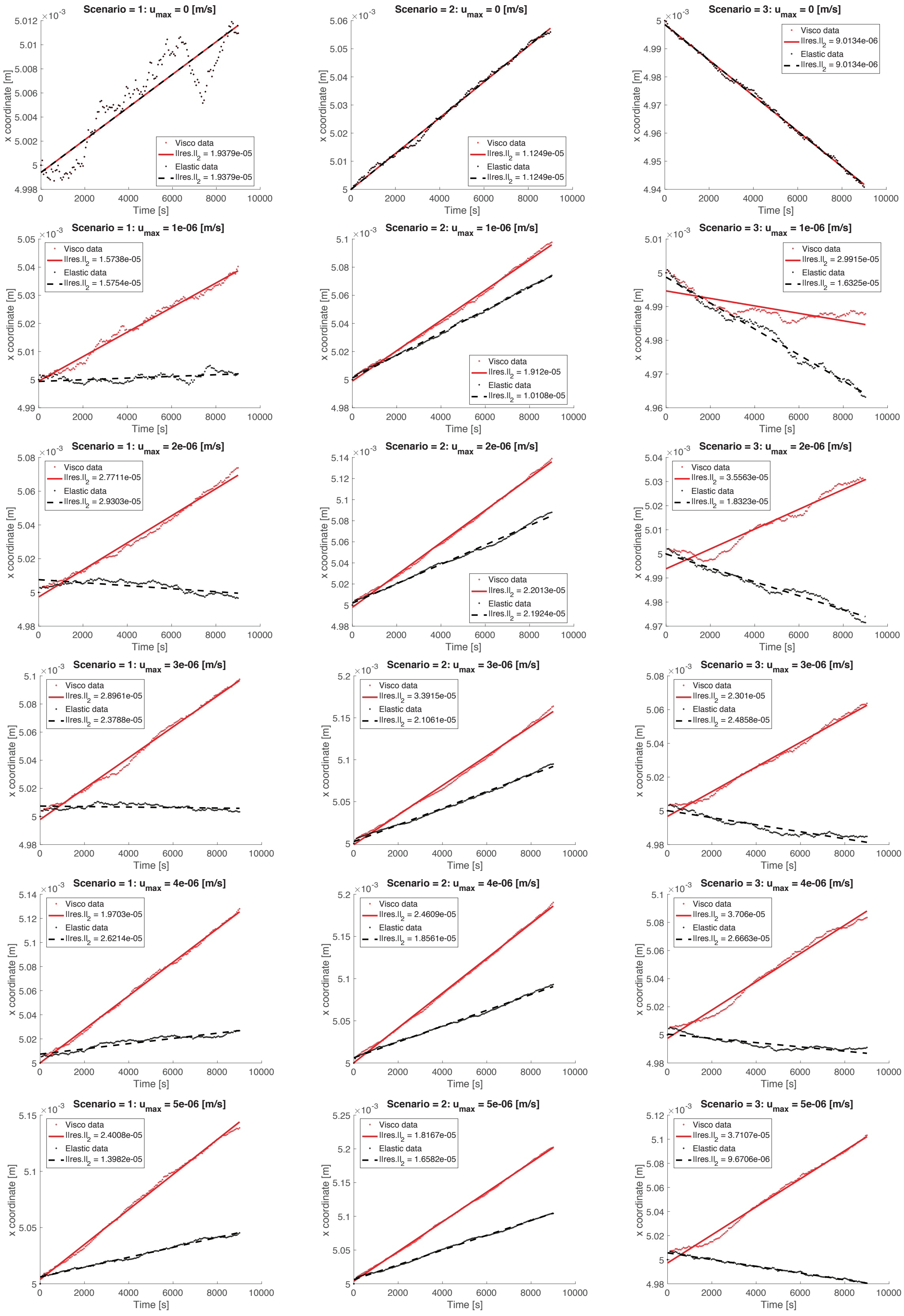

Supplementary Figure 5. Linear least squares fits to the x-coordinates of the center of mass in three nutrient configurations (Scenario = 1: full-stream; Scenario = 2: downstream; Scenario = 3: upstream) and six values of $u_{\max}$.

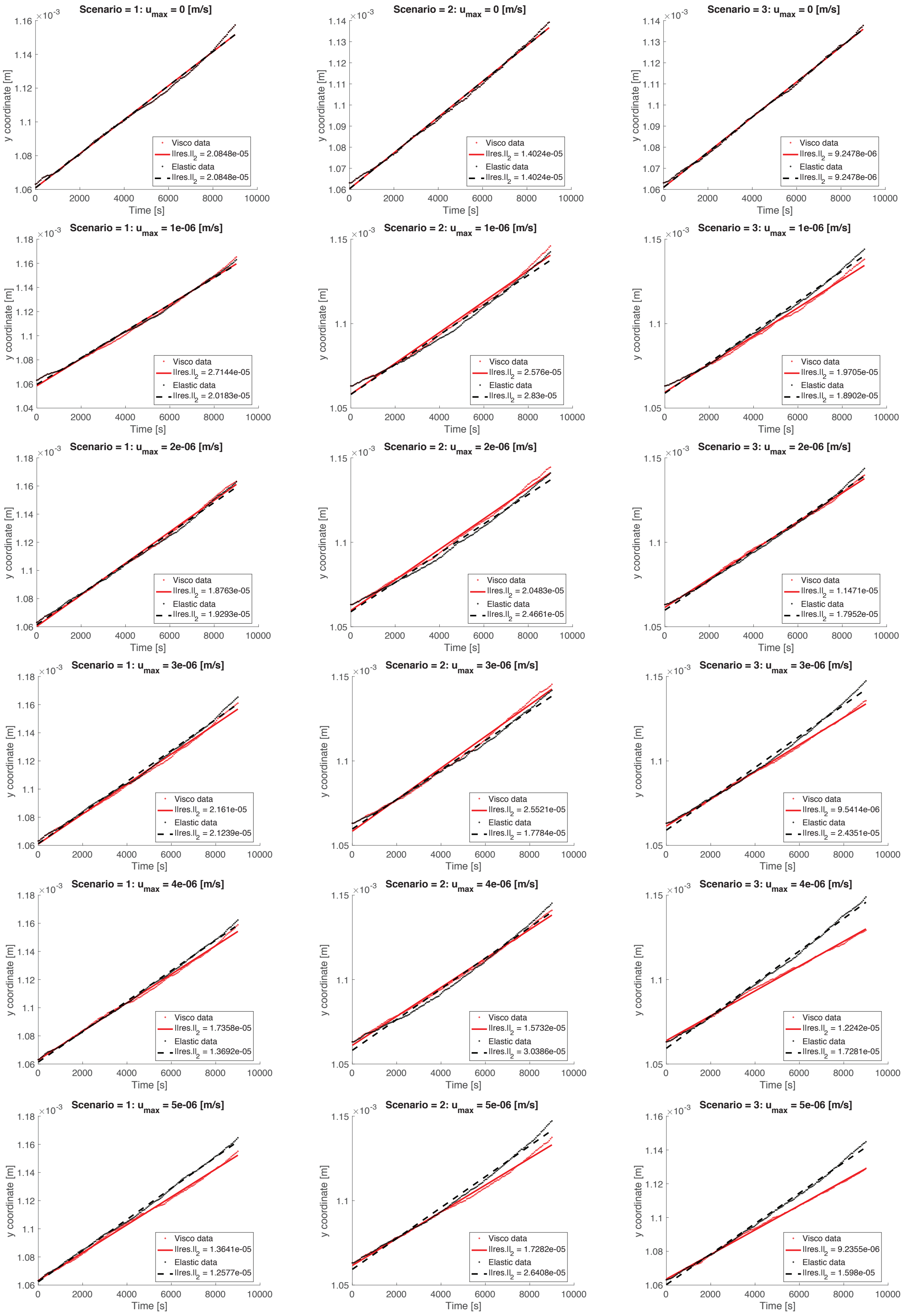

Supplementary Figure 6. Linear least squares fits to the y-coordinates of the center of mass in three nutrient configurations (Scenario = 1: full-stream; Scenario = 2: downstream; Scenario = 3: upstream) and six values of $u_{\max}$.

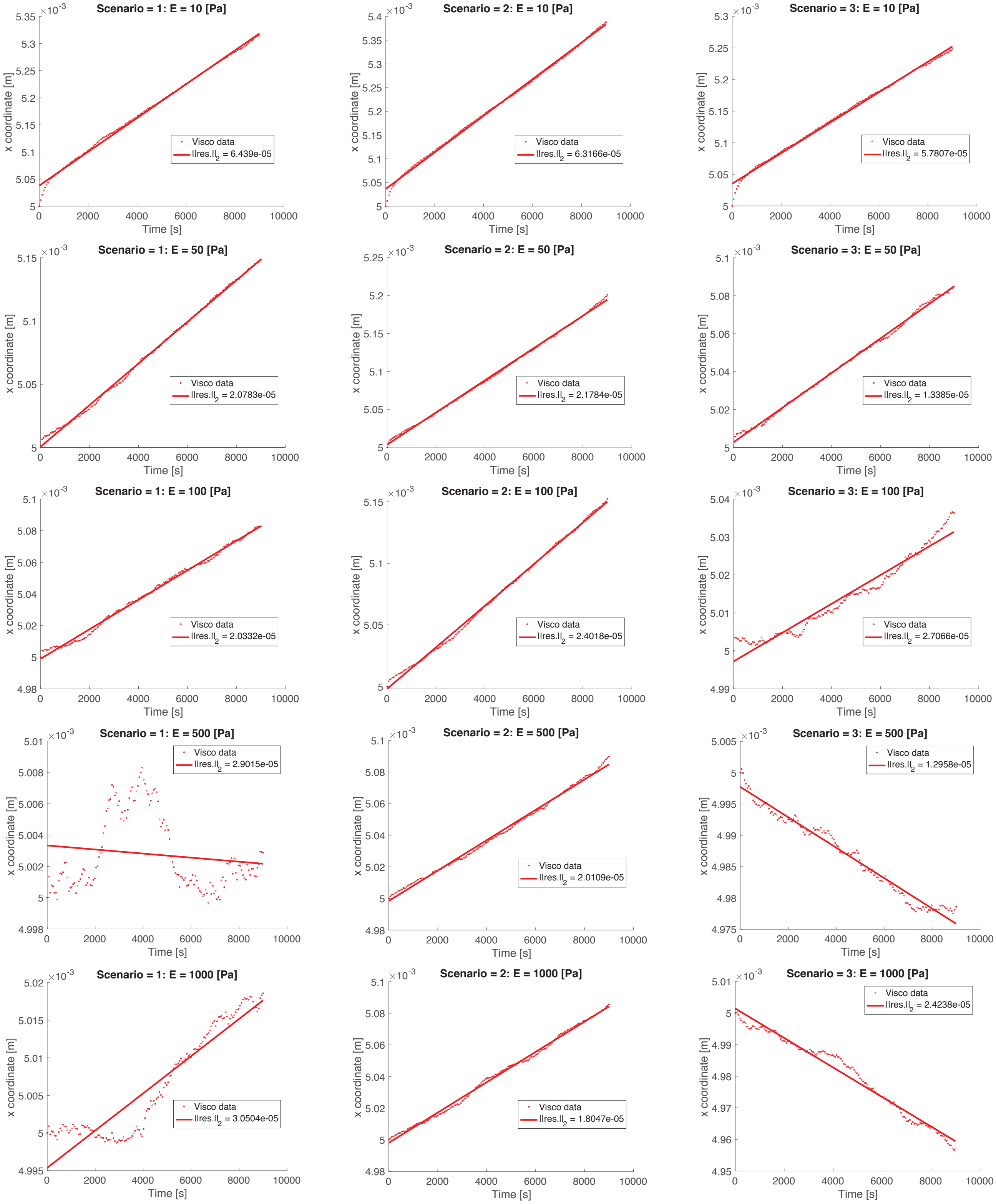

Supplementary Figure 7. Linear least squares fits to the x-coordinates of the center of mass in three nutrient configurations (Scenario = 1: full-stream; Scenario = 2: downstream; Scenario = 3: upstream) and five values of E.

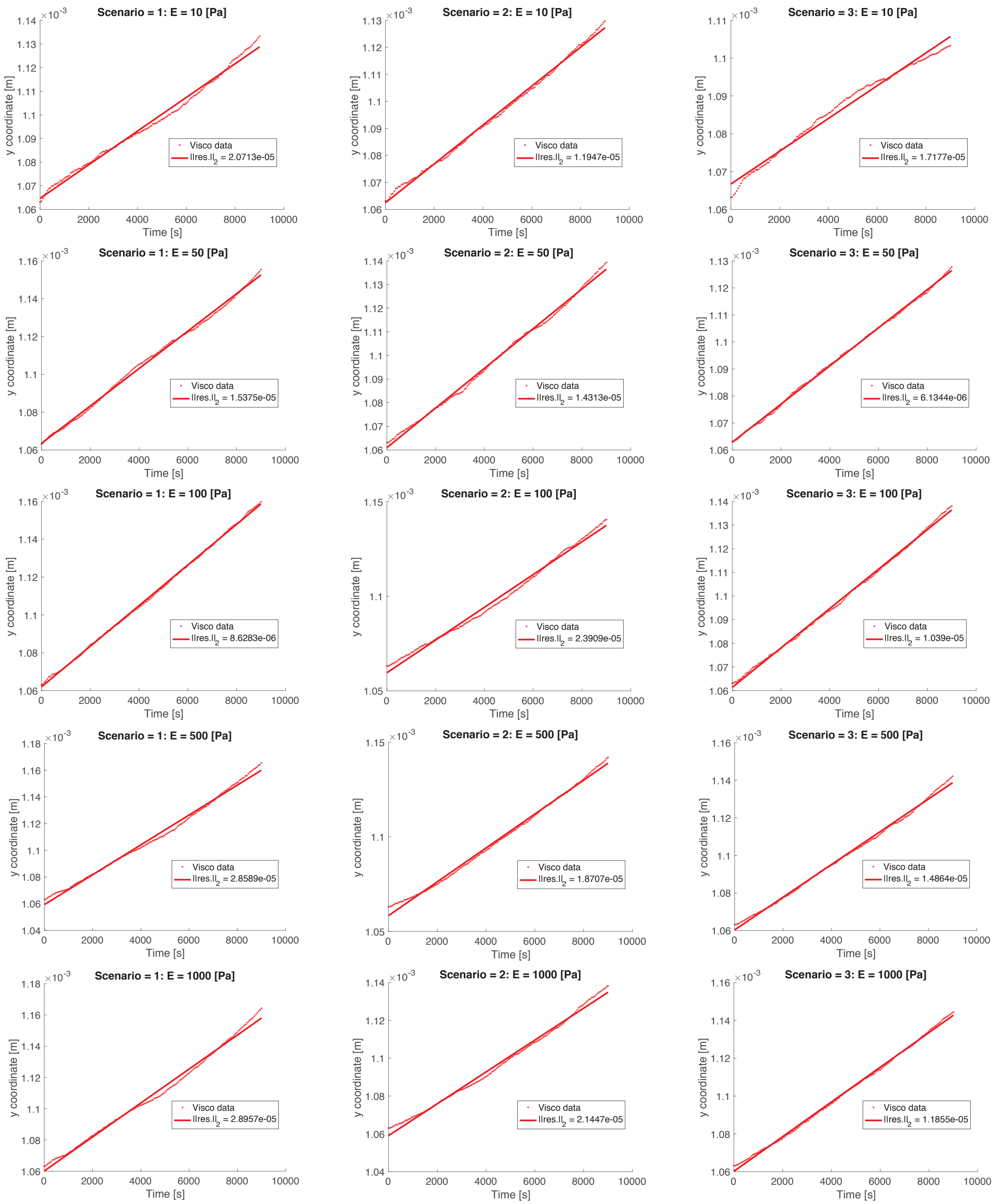

Supplementary Figure 8. Linear least squares fits to the *y*-coordinates of the center of mass in three nutrient configurations (Scenario = 1: full-stream; Scenario = 2: downstream; Scenario = 3: upstream) and five values of *E*.
