# Supplementary material for: Biofilm viscoelasticity and nutrient source location control biofilm growth rate, migration rate, and morphology in shear flow
Source: Sci Rep. 2021 Aug 9;11:16118. doi: 10.1038/s41598-021-95542-1 (PMC8352988; doi:10.1038/s41598-021-95542-1)
Supplement: Supplementary file 14 — Supplementary material 14. [file 41598_2021_95542_MOESM14_ESM.pdf]

Biofilm Viscoelasticity and Nutrient Source Location Control Biofilm Growth Rate, Migration Rate, and Morphology in Shear Flow

Hoa Nguyen[1], Abraham Ybarra[1], Hakan Başağaoğlu[2], and Orrin Shindell[3,*]

[1]Department of Mathematics, Trinity University, San Antonio, TX 78212, USA
[2]Evolution Online LLC, San Antonio, TX 78292, USA
[3,*]Department of Physics & Astronomy, Trinity University, San Antonio, TX 78212, USA
[*]oshindel@trinity.edu

The supplementary video files illustrate how the biofilm structures grow and deform under different nutrient source configurations and shear flow ($u_{\text{max}} = 5 \times 10^{-6}$ m/s) as time evolves. The first six videos reveal differences between a purely elastic biofilm and a biologically realistic viscoelastic biofilm. The last six videos show how the stiffness of the viscoelastic biofilm with relaxation time of 18 minutes affects its shape and growth as the biofilm interacts with the concentration field and shear flow in the surroundings. All these movies provide the time evolution of the biofilm formation behind some of the figures in the main text.
Our software package is available at Code Ocean (codeocean.com) for:
Creep test: https://doi.org/10.24433/CO.9059597.v1
Simulation of biofilm growth and deformation: https://doi.org/10.24433/CO.0816145.v1

Supplementary Video-1

`SI_Video1_1_Full_stream_elastic_u_5e_6.mp4`
Title: Dynamics of biofilm growth and deformation related to Fig. 3(b)
Legend: Elastic biofilm ($E = 50$ Pa) in the full-stream concentration configuration with the maximum shear flow $u_{\text{max}} = 5 \times 10^{-6}$ m/s

Supplementary Video-2

`SI_Video1_2_Full_stream_viscoelastic_u_5e_6.mp4`
Title: Dynamics of biofilm growth and deformation related to Fig. 3(f)
Legend: Viscoelastic biofilm ($E = 50$ Pa, $\eta = 5 \times 10^4$ Pa.s) in the full-stream concentration configuration with the maximum shear flow $u_{\text{max}} = 5 \times 10^{-6}$ m/s

Supplementary Video-3

`SI_Video1_3_Downstream_elastic_u_5e_6.mp4`
Title: Dynamics of biofilm growth and deformation related to Fig. 3(c)
Legend: Elastic biofilm ($E = 50$ Pa) in the downstream concentration configuration with the maximum shear flow $u_{\text{max}} = 5 \times 10^{-6}$ m/s

Supplementary Video-4

`SI_Video1_4_Downstream_viscoelastic_u_5e_6.mp4`
Title: Dynamics of biofilm growth and deformation related to Fig. 3(g)
Legend: Viscoelastic biofilm ($E = 50$ Pa, $\eta = 5 \times 10^4$ Pa.s) in the downstream concentration configuration with the maximum shear flow $u_{\text{max}} = 5 \times 10^{-6}$ m/s

Supplementary Video-5

SI_Video1_5_Upstream_elastic_u_5e_6.mp4
Title: Dynamics of biofilm growth and deformation related to Fig. 3(c)
Legend: Elastic biofilm ($E = 50$ Pa) in the upstream concentration configuration with the maximum shear flow $u_{\text{max}} = 5 \times 10^{-6}$ m/s

Supplementary Video-6

SI_Video1_6_Upstream_viscoelastic_u_5e_6.mp4
Title: Dynamics of biofilm growth and deformation related to Fig. 3(g)
Legend: Viscoelastic biofilm ($E = 50$ Pa, $\eta = 5 \times 10^4$ Pa.s) in the upstream concentration configuration with the maximum shear flow $u_{\text{max}} = 5 \times 10^{-6}$ m/s

Supplementary Video-7

SI_Video2_1_Full_stream_viscoelastic_u_5e_6_E_10.mp4
Title: Dynamics of biofilm growth and deformation related to Fig. 6(a)
Legend: Viscoelastic biofilm ($E = 10$ Pa, $\eta = 10290$ Pa.s) in the full-stream concentration configuration with the maximum shear flow $u_{\text{max}} = 5 \times 10^{-6}$ m/s

Supplementary Video-8

SI_Video2_2_Full_stream_viscoelastic_u_5e_6_E_500.mp4
Title: Dynamics of biofilm growth and deformation related to Fig. 6(d)
Legend: Viscoelastic biofilm ($E = 500$ Pa, $\eta = 539490$ Pa.s) in the full-stream concentration configuration with the maximum shear flow $u_{\text{max}} = 5 \times 10^{-6}$ m/s

Supplementary Video-9

SI_Video2_3_Downstream_viscoelastic_u_5e_6_E_10.mp4
Title: Dynamics of biofilm growth and deformation related to Fig. 6(e)
Legend: Viscoelastic biofilm ($E = 10$ Pa, $\eta = 10290$ Pa.s) in the downstream concentration configuration with the maximum shear flow $u_{\text{max}} = 5 \times 10^{-6}$ m/s

Supplementary Video-10

SI_Video2_4_Downstream_viscoelastic_u_5e_6_E_500.mp4
Title: Dynamics of biofilm growth and deformation related to Fig. 6(h)
Legend: Viscoelastic biofilm ($E = 500$ Pa, $\eta = 539490$ Pa.s) in the downstream concentration configuration with the maximum shear flow $u_{\text{max}} = 5 \times 10^{-6}$ m/s

Supplementary Video-11

SI_Video2_5_Upstream_viscoelastic_u_5e_6_E_10.mp4
Title: Dynamics of biofilm growth and deformation related to Fig. 6(i)
Legend: Viscoelastic biofilm ($E = 10$ Pa, $\eta = 10290$ Pa.s) in the upstream concentration configuration with the maximum shear flow $u_{\text{max}} = 5 \times 10^{-6}$ m/s

Supplementary Video-12

SI_Video2_6_Upstream_viscoelastic_u_5e_6_E_500.mp4
Title: Dynamics of biofilm growth and deformation related to Fig. 6(l)
Legend: Viscoelastic biofilm ($E = 500$ Pa, $\eta = 539490$ Pa.s) in the upstream concentration configuration with the maximum shear flow $u_{\text{max}} = 5 \times 10^{-6}$ m/s

Acknowledgements

This research was supported by NSF DMS-1720323 to HN, HB, and AY and NSF MRI-1531594 to HN. We thank Trinity University for the Summer Research Grant to OS and the Mach Fellowship to AY, and the provision of computational resources.
